# Type 2 Diabetes Mellitus and Clinicopathological Tumor Characteristics in Women Diagnosed with Breast Cancer: A Systematic Review and Meta-Analysis

**DOI:** 10.3390/cancers13194992

**Published:** 2021-10-05

**Authors:** Fan Zhang, Jing de Haan-Du, Grigory Sidorenkov, Gijs W. D. Landman, Mathilde Jalving, Qingying Zhang, Geertruida H. de Bock

**Affiliations:** 1 Department of Epidemiology, University Medical Center Groningen, University of Groningen, 9713 GZ Groningen, The Netherlands; f.zhang@umcg.nl (F.Z.); j.du@umcg.nl (J.d.H.-D.); g.h.de.bock@umcg.nl (G.H.d.B.); 2 Department of Preventive Medicine, Shantou University Medical College, Shantou 515041, China; qyzhang@stu.edu.cn; 3 Guangdong Provincial Key Laboratory for Breast Cancer Diagnosis and Treatment, Cancer Hospital of Shantou University Medical College, Shantou 515041, China; 4 Department of Internal Medicine, Gelre Hospital, 7334 DZ Apeldoorn, The Netherlands; g.landman@gelre.nl; 5 Department of Oncology, University Medical Center Groningen, University of Groningen, 9713 GZ Groningen, The Netherlands; m.jalving@umcg.nl

**Keywords:** type 2 diabetes mellitus, breast cancer, tumor stage, tumor size, lymph node status

## Abstract

**Simple Summary:**

Female breast cancer continues to be the leading cause of cancer deaths worldwide, and type 2 diabetes mellitus (T2DM) is one of the contributors to the poor prognosis of breast cancer. This raises the issue that T2DM might be associated with aggressive clinicopathological characteristics, which indicate pivotal prognostic values. This study aimed to clarify the differences in breast cancer characteristics at diagnosis between patients with and without pre-existing T2DM. Our meta-analyses showed an increased risk of being diagnosed with a late-stage tumor, large tumor size, and invasive lymph nodes in patients with T2DM. No significant results were observed for grade, estrogen/progesterone receptor, and human epidermal growth factor receptor. These findings indicate an association between T2DM and advanced breast cancer at diagnosis, and suggest that the more active role of breast cancer screening should be further explored for women with T2DM.

**Abstract:**

Poor prognosis caused by type 2 diabetes mellitus (T2DM) in women with breast cancer is conferred, while the association between T2DM and breast tumor aggressiveness is still a matter of debate. This study aimed to clarify the differences in breast cancer characteristics, including stage, size, lymph node status, grade, estrogen receptor (ER), progesterone receptor (PR), and human epidermal growth factor receptor (Her2), between patients with and without pre-existing T2DM. PubMed, Embase, and Web of Science were searched for studies from 1 January 2010 to 2 July 2021. Adjusted odds ratios (ORs) with 95% confidence intervals (CIs) were pooled by using a random effects model. T2DM was significantly associated with tumor stages III/IV versus cancers in situ and stages I/II (pooled ORs (pOR), 95% CI: 1.19; 1.04–1.36, *p* = 0.012), tumor size >20 versus ≤20 mm (pOR, 95% CI: 1.18; 1.04–1.35, *p* = 0.013), and lymph node invasion versus no involvement (pOR, 95% CI: 1.26; 1.05–1.51, *p* = 0.013). These findings suggest that women with T2DM are at a higher risk of late-stage tumors, large tumor sizes, and invasive lymph nodes at breast cancer diagnosis.

## 1. Introduction

Global breast cancer incidence has been increasing during the last three decades [1]. Female breast cancer has overtaken lung cancer as the most commonly diagnosed cancer, with a total of 2.26 million cases in the year 2020 [2]. Despite the steadily decreasing mortality rate of breast cancer in high-resource countries [3], breast cancer remains as the leading cause of cancer deaths and disability-adjusted-life-years for women worldwide [4]. It is estimated that more than one million women will die of breast cancer in the year 2040 [5].

Tumor stage at diagnosis is one of the key characteristics that defines breast cancer prognosis. The mortality risk assessed by hazards ratios rises with the increasing tumor stage and is 10-fold higher in stages III and IV compared to stage I [6,7,8,9]. Histologic grade, determined by morphologic features, can accurately predict tumor behavior, particularly in earlier small tumors [10]. High-grade breast cancers tend to recur and metastasize early following diagnosis, in contrast to those at low grade [10,11]. The molecular features, namely hormone receptors (i.e., estrogen receptor (ER) and progesterone receptor (PR)) and human epidermal growth factor receptor 2 (Her2), are useful for defining subtypes and guiding therapeutic choices [12]. Hormone receptors+/Her2- subtype takes up 70% and possesses a highest survival rate, followed by Her2+ subtype and triple-negative subtype (TNBC) [13,14]. Owing to cancer screening, women tend to be diagnosed with early stage [15], well-differentiated [16], and hormone-receptors-positive breast tumors [17,18]. 

Type 2 diabetes mellitus (T2DM) is another serious public health concern worldwide [19], showing increasing global prevalence that was ~9% in female adults in 2019. Associations between T2DM and breast cancer have been intensively studied, particularly about diabetes and the risk, as well as the prognosis of breast cancer. According to previous systematic evidence, diabetes is associated with a 14% to 25% increased risk of breast cancer [20,21,22], and with a 37% to 61% elevated hazard of all-cause mortality in breast cancer patients [23,24,25]. Next to a lower participation rate in screening and delayed detection [26], the burden of diabetes on the poor prognosis of patients with breast cancer could be due to metabolic abnormalities. In vitro experiments found that hyperglycemia and hyperinsulinemia could accelerate proliferation and migration of breast cancer cells [27,28], potentially resulting in aggressive breast tumor characteristics at diagnosis (e.g., large tumor size, lymph node metastasis, and late-tumor stage). However, evidence of the associations between T2DM and aggressive clinicopathological characteristics of breast cancer at diagnosis still remains inconclusive in epidemiological studies. 

To date, as far as we know, there are only two reviews summarizing the relation between diabetes and breast cancer characteristics based on epidemiological studies. One of the studies, which had a literature search until July 2009, briefly suggested that pre-existing diabetes might be associated with a later stage of breast cancer at diagnosis [29]. However, this review still lacks quantitative evidence, and more importantly, it was just based on four articles and their crude estimates, which were not adjusted for potential confounders. The other review with a literature search until December 2018 suggested that diabetes significantly increased the risk of lymph node metastasis [30]; however, this statement is made without considering adjustment, either. Therefore, the evidence that diabetes is associated with an increased risk of being diagnosed with more aggressive breast cancer is unconvincing; confounding by other factors cannot be excluded. 

In this systematic review and meta-analysis, we aimed to update the evidence based on adjusted estimates and quantitatively analyze the differences in tumor stage and lymph node status at breast cancer diagnosis between patients with and without pre-existing T2DM. Furthermore, as far as we know, our review firstly outlined the associations between diabetes and the following breast cancer characteristics: tumor size, grade, estrogen receptor (ER), progesterone receptor (PR), human epidermal growth factor receptor (Her2), and molecular subtypes between patients with and without pre-existing T2DM. The results could support the formulation of preventive measures and screening strategies for women with diabetes.

## 2. Materials and Methods

This study was conducted based on a registered protocol (the International Prospective Register of Systematic Reviews, registration number CRD42020208704, http://www.crd.york.ac.uk/PROSPERO/, accessed on 17 October 2020) and reported according to the Preferred Reporting Items for Systematic Reviews and Meta-Analyses (PRISMA) guidelines [31].

### 2.1. Exposure and Outcomes

The exposure of interest was pre-existing T2DM at breast cancer diagnosis. The primary outcome was tumor stage, and the secondary outcomes were tumor size, lymph node status, histological grade, ER, PR, Her2 status, and molecular subtypes. 

### 2.2. Systematic Search

A systematic database search in PubMed, Embase and Web of Science was conducted. In the previous review with a literature search from the inception to July 2009 [29], four studies were available on the association between diabetes and tumor stage, but only crude estimates were accessible; therefore, we updated the evidence from 1 January 2010 to 2 July 2021. Search strings included MeSH terms and free text words on (1) breast cancer (e.g., breast tumor and breast carcinoma), (2) diabetes, and (3) tumor characteristics (e.g., staging, lymph node status, grade, size, ER, PR, and Her2) (Materials S1). 

### 2.3. Study Selection

Articles were eligible for this review if they met the following criteria: (1) evaluated a population of women diagnosed with primary breast cancer; (2) confirmed diabetes status before or at breast cancer diagnosis, or before any anticancer treatment; (3) evaluated prespecified tumor characteristics of breast cancer by diabetes status; and (4) reported adjusted risk estimates (e.g., odds ratios (ORs)) or provided sufficient data for calculating adjusted ORs (i.e., a paired study design matching at least for age), which demonstrated associations between diabetes and breast tumor characteristics. No restrictions for study design were applied. Exclusion criteria were (1) other type of diabetes than T2DM, such as type 1 diabetes mellitus (T1DM) and gestational diabetes; (2) participants were mainly younger than 30 years old if type of diabetes was not clearly specified; (3) full text was not available (e.g., conference abstracts); and (4) languages other than English, Dutch, or Chinese. In case of multiple publications on the same cohort, the results based on the largest sample size were selected.

### 2.4. Data Extraction

Titles, abstracts, and full texts were reviewed by two independent reviewers (F.Z. and J.d.H.-D.), and the following data were extracted: study design, region, study participants (e.g., age at breast cancer diagnosis, menopausal status, and calendar year of breast cancer diagnosis), sample size, ascertainment of diabetes, and breast tumor characteristics, as well as the corresponding classification system, statistical methods, and results in the associations between T2DM and breast cancer characteristics. Whenever possible, adjusted risk estimates were used for the meta-analyses. As for the articles with a matched study design, ORs were calculated based on the frequency tables. 

### 2.5. Methodological Quality Assessment

Diabetes was suspected to be a prognostic factor for developing breast cancer with aggressive tumor characteristics; therefore, the Quality of Prognosis Studies (QUIPS) tool [32] was applied and adjusted to the present research questions. Two reviewers (F.Z. and J.d.H.-D.) independently assessed the methodological quality of individual studies (Materials S2). Disagreements were resolved in consensus meetings, and a third reviewer (G.S.) made a final decision in case of persistent disagreements. There are six domains criticizing potential risk of bias (ROB): study participation, attrition, diabetes measurement, measurement of tumor characteristics, study confounding, and statistical analysis and reporting. The “attrition” domain, which assumes longitudinal study design, is inapplicable for cross-sectional or case-control studies, and was only assessed for nested case-control studies [33,34]. As we considered age and body mass index (BMI) to be important confounders in the relation between T2DM and breast cancer clinicopathological characteristics [35,36,37,38], regarding the domain of study confounding, articles adjusting for both of these two factors were considered as having low ROB. Considered that adequate description of participants’ selection was crucial for our research question, “study participation” (bias domain 1) was selected as the most important domain when assessing the overall quality for each study [32]. The overall quality of a study was considered to be (1) high if ≤2 domains were rated a moderate ROB and all the others, including the domain “study participation”, were rated a low ROB; (2) low if ≥1 domain was rated a high ROB, irrespective of all the other domains; and (3) moderate in other situations [39,40]. The overall percentage of agreement and Cohen’s kappa were calculated to evaluate inter-rater agreement.

### 2.6. Statistical Analysis

A random effects model with the inverse variance method was employed to combine individual adjusted estimates to obtain pooled ORs (pORs), and the between-study variance was estimated by the “Sidik–Jonkman” method [41]. To maximize the sample sizes in the meta-analysis, we chose frequently used and clinically relevant cutoffs for the primary analysis and performed sensitivity analyses where possible. According to the National Cancer Institute Dictionary of Cancer Terms (https://www.cancer.gov/, accessed on 9 September 2021), early stage breast cancers are those constrained in the breast or the axillary lymph nodes (i.e., stage I, stage IIA, stage IIB, and stage IIIA based on the TNM staging system). Considering the available datasets, here we defined the early stage to be those cancers in situ or at stages I/II, and late stage to be those at stages III/IV. With regard to the other staging system of invasive tumors used in the included papers, tumors were grouped into localized (constraint to breast tissue), regional (direct extension and/or involvement of lymph nodes) tumors, and distant metastases. ORs of distant metastases/regional tumors versus localized tumors were pooled with ORs of stages III/IV versus cancers in situ/stages I/II in the meta-analysis. As for the secondary outcomes, the comparisons were tumor size “>20 mm” versus “≤20 mm”, lymph node status “positive” versus “negative”, histological grades “2/3” versus grade “1”, ER (or PR) status “negative” versus “positive”, Her2 status “positive” versus “negative” and molecular subtype “TNBC” versus “others”. Since only a few papers clarified the cutoffs and techniques for ER/PR/Her2 detection and classification [42,43,44,45], we followed the division of their expression status in the individual articles. For the studies with unexpected cutoffs (e.g., stage I was in the reference group, while stages II, III, and IV were separately treated as different levels of outcomes) or inverse association, transformation [46,47] was conducted to facilitate the pooling when necessary (Materials S3). Heterogeneity was examined by using the Q-test and the *I*^2^ index. The *p*-value from Q-test (*P*_Q_) < 0.05 qualitatively suggested heterogeneous results in the pooled analysis. Publication bias was assessed by plotting funnel plots and tested for asymmetry by Egger’s method at *p* < 0.10 level [48]. All statistical analyses were performed in R (version 4.0.3) with the package “meta”.

### 2.7. Stratified Analyses and Sensitivity Analyses

In case of a considerable heterogeneity (*I*^2^ ≥ 75%), no pooled estimate would be calculated; in case of a moderate (*I*^2^ ≥ 30%) or a substantial heterogeneity (*I*^2^ ≥ 50%) [49], stratified analyses by the following variables were planned when two or more studies were available in each category: (1) characteristics of the study population (i.e., region, calendar year of breast cancer diagnosis, sample size, and age at breast cancer diagnosis). As for calendar year of breast cancer diagnosis, a median ≤2008 was used to identify articles recruiting participants diagnosed in the early days. Regarding age at breast cancer diagnosis, patients at pre-menopausal status (or ≤50 years old) were deemed to be young, whereas others at post-menopausal status (or >50 years old) were elderly patients. Then studies were classified based on the proportion of young participants ≤25% or >25%; (2) adjustment for possible confounders (i.e., BMI, socioeconomic status (SES), comorbidity score, and breast cancer screening), due to the possible confounding effect of these factors in the relationship between diabetes and breast tumor characteristics [50,51,52,53]; (3) ROB of the study participation domain; and (4) the overall quality based on ROB. As for tumor stage, stratified analysis was also conducted by different staging systems. Due to insufficient number of studies in each subgroup, tests (e.g., Q test [54]) are inappropriate to assess subgroup differences of pORs. Instead, if the confidence interval of one subgroup failed to cover the point estimate of the pOR in the other subgroup, we considered this stratified factor partly responsible for the heterogeneity.

Influence analyses (i.e., leave-one-out sensitivity analysis) were performed by omitting one study at a time. Sensitivity analyses were performed for: (1) tumor stage (a) by comparing stage IV to cancers in situ and stages I/II/III (distant metastases versus localized/regional tumors), (b) by excluding articles with cancers in situ, or (c) by excluding articles without distant metastasis (i.e., tumors at stage IV); (2) tumor size by setting the cutoff to be 50 mm instead of 20 mm; (3) lymph node status by comparing N2/N3 to N0/N1; and (4) tumor grade by comparing grade 3 versus grades 1 and 2. 

## 3. Results

### 3.1. Description of the Included Studies

**Figure 1:**
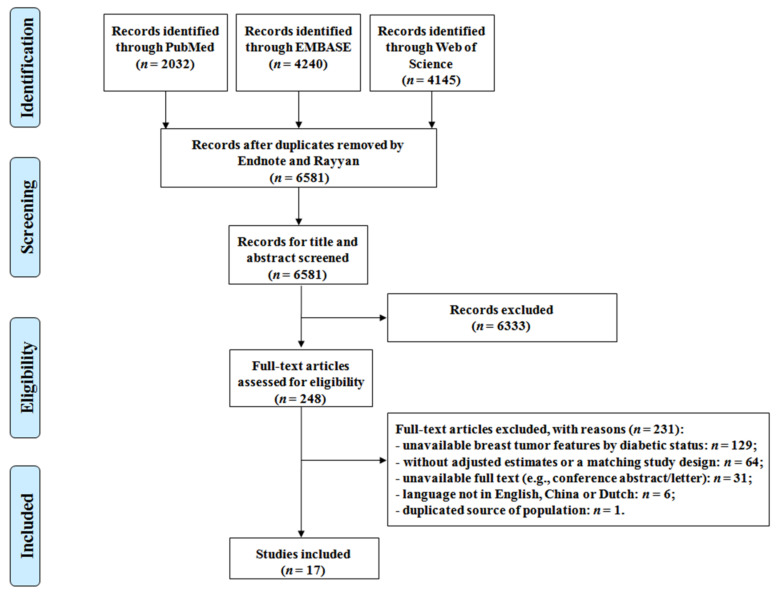
Flow diagram of included studies.

Of the 6581 identified studies, 248 were reviewed for full-text, and 17 were included in this systematic review (Figure 1) [42,43,44,45,55,56,57,58,59,60,61,62,63,64,65,66,67]. The study characteristics and extracted data are presented in Table 1 and Table S1. A majority of the included studies (15 out of 17) [42,43,44,45,55,58,59,60,61,62,63,64,65,66,67] were cross-sectional; one was a nested case-control study [56], and one was a case-control study [57]. More than half of the studies were conducted in the Western countries (10 out of 17) [43,55,56,57,59,61,62,64,65,66], and the others were performed in the Eastern countries [42,44,45,58,60,63,67]. Ten studies enrolled patients diagnosed with breast cancer in the early days (a median calendar year of breast cancer diagnosis ≤2008) [42,43,45,56,59,60,61,65,66,67], while the other recruited patients lately [44,55,57,58,62,63,64]. The majority of articles had a low proportion of young participants (≤25%) at breast cancer diagnosis [45,55,56,59,60,62,65,66]. Only six studies enrolled a relatively young population [42,43,57,61,63,67], and in in three studies, the % of young participants could not be determined, due to insufficient information [44,58,64].

Study populations were either from hospitals [42,44,45,58,60,63,64,66,67] or registries [43,55,56,57,59,61,62]. Study size ranged from 20 to 6115 breast cancer patients with diabetes, and from 20 to 67,701 breast cancer patients without diabetes. Various methods were used to ascertain T2DM in patients, i.e., based on medical records, antidiabetic drugs used by self-reports or insurance claims, and/or FPG (fasting plasma glucose) or HbA1c (hemoglobin A1c) levels above thresholds (Table S1). With regard to the primary outcome, breast cancer stage was either reported based on TNM classification [45,55,56,58,62,64,66] or invasive tumor categories: localized, regional tumors, and distant metastases [59,61].

### 3.2. Risk of Bias (ROB) of Eligible Studies

Results of the methodological quality assessment are presented in Table S2. Notably, of all the 17 eligible studies, only three [43,56,59] showed an overall low ROB, whilst the majority [42,44,45,55,57,58,60,61,62,63,64,65,66,67] exhibited a moderate or high ROB. A high risk of bias was most often assigned to the domain “Study Participations” (in six studies) [58,60,61,63,64,67], mainly due to poor description of participants and the inclusion criteria, and then the domain “Determinant Measurement” (in three studies) [42,45,65], because of the unreliable measurement or selection bias in diabetes population (e.g., one-time FBG measurement [42], or defining T2DM by metformin use only [45,65]). The two domains “Outcome Measurements” and “Statistical Analysis and Reporting” were less likely biased (low risk ratings in 14 [42,43,44,45,55,57,58,59,60,61,62,64,66] and 13 articles [43,44,45,55,56,57,59,60,61,62,63,64,66], respectively). The inter-rater agreement on the internal validity domains was good (overall agreement, 91.9% (79/86); Cohen’s kappa, 0.87).

### 3.3. Associations between T2DM and Tumor Characteristics

#### 3.3.1. Primary Outcome

**Figure 2:**
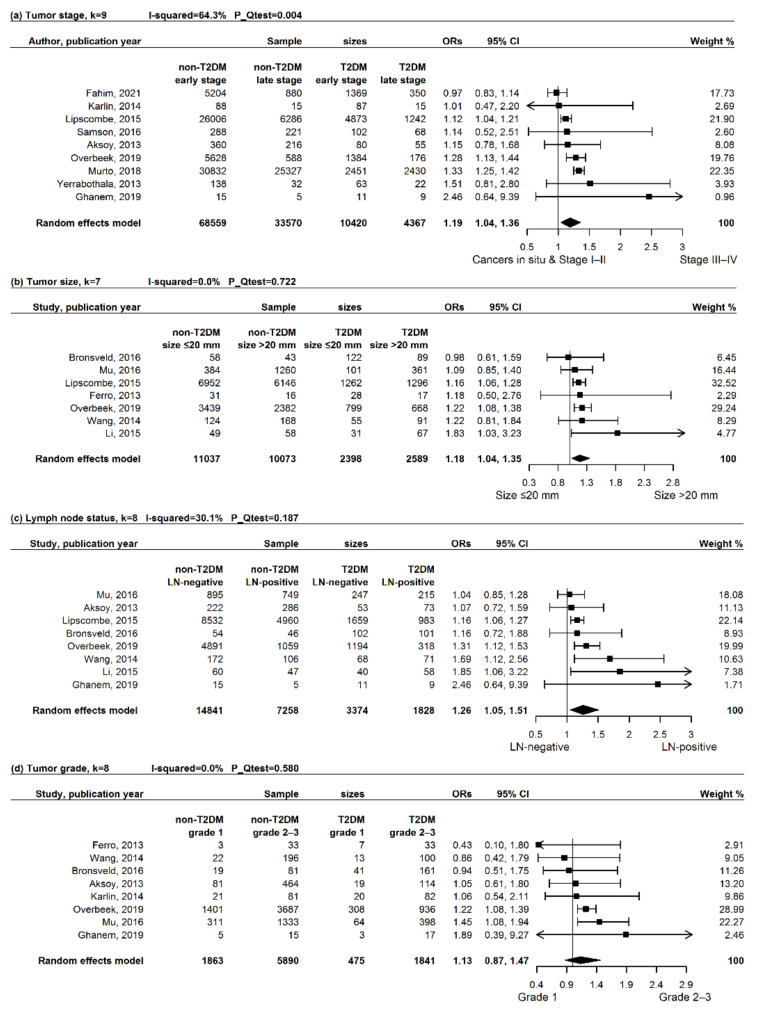

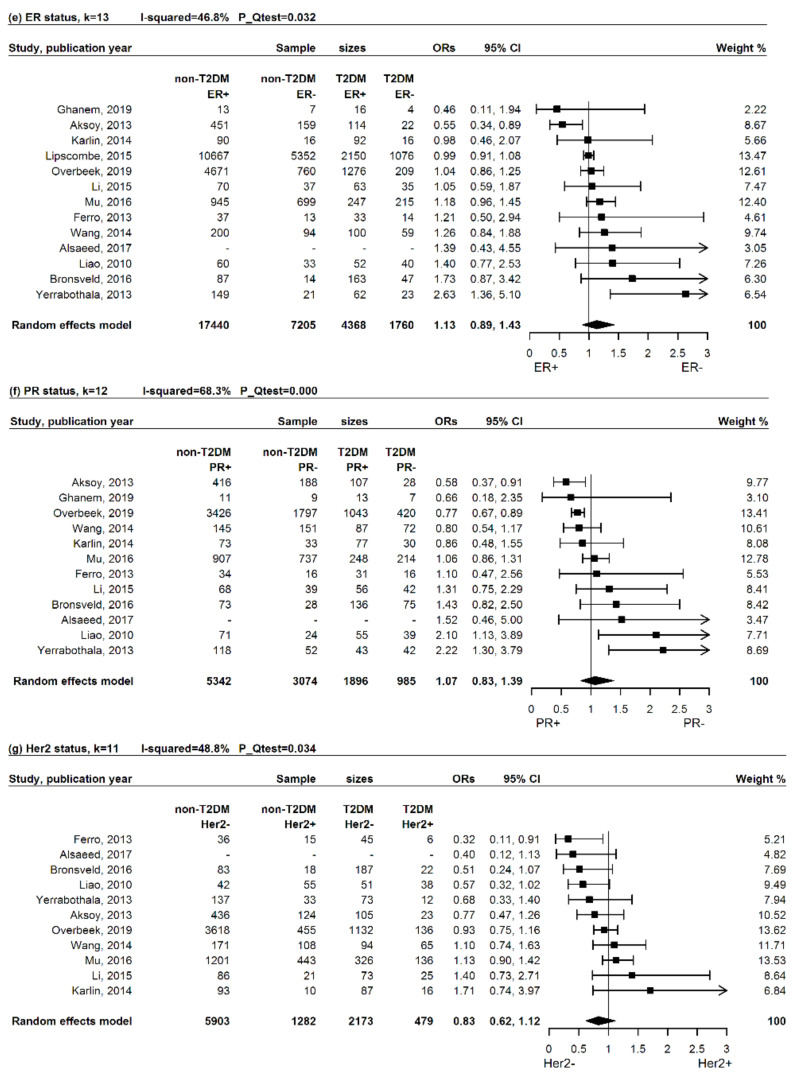
Forest plots of the associations between T2DM and tumor characteristics at diagnosis of breast cancer. Forest plots were created for the associations between T2DM and (**a**) tumor stage, (**b**) tumor size, (**c**) lymph node status, (**d**) tumor grade, (**e**) ER status, (**f**) PR status, and (**g**) Her2 status. As for the article “Alsaeed, 2017”, sample sizes of patients with and without diabetes by ER/PR/Her2 status were unavailable; therefore, the total sample sizes did not include the population in this study. T2DM, type 2 diabetes mellitus; LN, lymph node; ER, estrogen receptor; PR, progesterone receptor; Her2, human epidermal growth factor receptor-2; ORs, odds ratio; CI, confidence interval; P_Qtest, *p*-value from Q-test.

**Figure 3:**
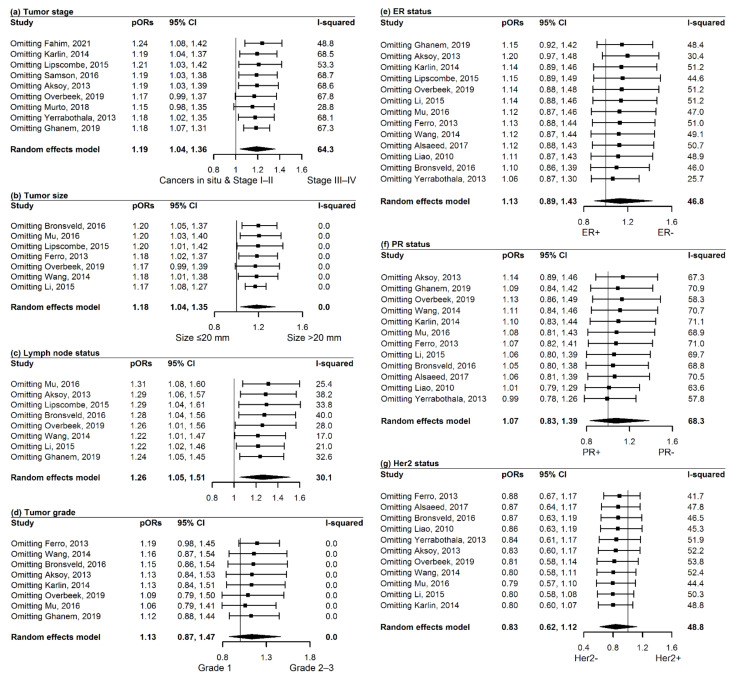
Influence analyses for examining impact of each article on the pooled estimates. Influence analyses were conducted for (**a**) tumor stage; (**b**) tumor size, (**c**) lymph node status, (**d**) tumor grade, (**e**) ER status, (**f**) PR status, and (**g**) Her2 status. pORs, pooled odds ratio; CI, confidence interval; ER, estrogen receptor; PR, progesterone receptor; Her2, human epidermal growth factor receptor-2.

Nine articles [45,55,56,58,59,61,62,64,66] reported tumor stage, and 14,787 (range: 20 to 6115) breast cancer patients in total were diagnosed with diabetes while 102,129 counterparts (range: 20 to 56,159) were without diabetes. As shown in Figure 2, at breast cancer diagnosis, patients with T2DM had an increased risk of being diagnosed with stages III/IV (versus cancers in situ and stages I/II), in contrast to counterparts without T2DM (pOR: 1.19, 95% CI: 1.04 to 1.36, *p* = 0.012). A substantial heterogeneity was observed (*I*^2^ = 64.3%, *P*_Q_ = 0.004), and none of the individual predefined stratification variables could explain that (Figure S1). Influence analyses showed that, when excluding either of the two articles [56,59], pORs were slightly reduced, and became insignificant (Figure 3). Sensitivity analyses all revealed comparable results with the primary analysis (Table S3).

#### 3.3.2. Secondary Outcomes: Tumor Size, Lymph Node Status, and Tumor Grade

Pooling estimates generated significant associations of diabetes with tumor size > 20 mm versus ≤20 mm (pOR, 95% CI: 1.18, 1.04 to 1.35, *p* = 0.013), and with lymph node invasion (pOR, 95% CI: 1.26, 1.05 to 1.51, *p* = 0.013), but not with tumor grades 2 and 3 versus grade 1 (pOR, 95% CI: 1.13, 0.87 to 1.47, *p* = 0.352, Figure 2). No stratified analysis was conducted for tumor size (*I*^2^ = 0%, *P*_Q_ = 0.722) and tumor grade (*I*^2^ = 0%, *P*_Q_ = 0.580) because of non-observed heterogeneity. Stratified analyses failed to identify distinct factors for the moderate heterogeneity in the pooled estimate of lymph node status (*I*^2^ = 30.1%, *P*_Q_ = 0.187; Figure S2).

Influence analyses did not show exceptional impact of included articles on the pOR for tumor size and lymph node status, but revealed a possible impact of the article [65] on the overall pORs for tumor grade. Exclusion of this article led to a non-significant but marginal result that T2DM was associated with a high level of tumor grade (pOR, 95% CI: 1.19, 0.98 to 1.45, *p* = 0.074, Figure 3). Sensitivity analyses found insignificant associations of T2DM with these three tumor characteristics (Table S3).

#### 3.3.3. Secondary Outcomes: ER, PR, Her2, and TNBC

Meta-analyses all yielded insignificant pooled estimates of the associations between diabetes and ER/PR/Her2 status (pOR, 95% CI for ER negativity versus ER positivity: 1.13, 0.89 to 1.43, *p* = 0.310; PR negativity versus PR positivity: 1.07, 0.83 to 1.39, *p* = 0.595; Her2 positivity versus Her2 negativity: 0.83, 0.62 to 1.12, *p* = 0.232, Figure 2). A moderate/substantial heterogeneity was separately found in these three pORs (*I*^2^ = 46.8%, 68.3%, and 48.8%, *P*_Q_ = 0.032, <0.001, and 0.034). Stratified analyses did not find explanation for the heterogeneity in the pOR of ER (Figure S3). Otherwise, age at breast cancer diagnosis and adjustment for SES were possibly in part responsible for the heterogeneity in the pOR of PR (Figure S4), as well as that the calendar year of breast cancer diagnosis and BMI possibly in part interpreted the heterogeneity in the pOR of Her2 (Figure S5). With regard to PR, a stronger pOR was observed in the subgroup with a proportion of young patients >25% (pOR, 95% CI: 1.55, 1.10 to 2.17, *p* = 0.011) than that in the other subgroup with elderly patients (pOR, 95% CI: 1.00, 0.65 to 1.54, *p* = 0.997). In two studies adjusting for SES [56,58], the pOR of PR significantly indicated a reverse association (pOR, 95% CI: 0.77, 0.66 to 0.89, *p* < 0.001), which was not seen in the articles without adjustment for SES (pOR, 95% CI: 1.15, 0.87 to 1.52, *p* = 0.326). Regarding Her2, patients with T2DM were less likely to develop Her2-positive tumors, this was reported in studies with patients diagnosed in the early days (pOR, 95% CI: 0.73, 0.54 to 0.98, *p* = 0.036), but not in later studies (pOR, 95% CI: 1.25, 0.89 to 1.77, *p* = 0.201); this was also seen in studies adjusting BMI (pOR, 95% CI: 0.47, 0.25 to 0.88, *p* = 0.019), but not in those without adjustment for BMI (pOR, 95% CI: 0.91, 0.67 to 1.23, *p* = 0.537). Influence analyses separately showed similar effect of the included articles on the pooled estimates for PR and Her2, but indicated a plausible impact of the article [45] on the pOR of ER. Exclusion of this article led to an insignificant but stronger association of T2DM with ER-negativity (pOR, 95% CI: 1.20, 0.97 to 1.48, *p* = 0.097, Figure 3).

There were only five papers [43,45,56,57,66] exploring the association between diabetes and molecular subtypes (TNBC or not). A considerable heterogeneity was found (*I*^2^ = 79.3%, *P*_Q_ = 0.001); therefore, individual results were not pooled. Two of these studies suggested a positive relation between diabetes and TNBC [57,66], but this was not supported by results of the other study that showed an inverse association [45], and the remaining two papers indicated no association [43,56].

#### 3.3.4. Publication Bias

Visual inspection of the funnel plots of included studies indicated improbable publication bias for all outcomes (Figure S6). Results of the Egger’s tests were in line with the visual inspection (all *p* > 0.1). 

## 4. Discussion

### 4.1. Summary of the Pooled Results

Our systematic review is the first meta-analysis that quantified the association between diabetic status and tumor characteristics, namely tumor size, grade, ER, PR, and Her2 status in women diagnosed with breast cancer, on the basis of epidemiological studies. We observed a significant association between T2DM and large tumor size. Although the effect estimates pointed to a higher risk in women with T2DM for high-grade tumor and ER/PR/Her-negativity, in comparison to non-diabetic counterparts, these results did not meet the statistical significance. No conclusion could be made for TNBC, because of a limited number of studies and a considerable heterogeneity.

In addition, we updated the evidence with the most recent articles and used estimates adjusted for potential confounders, and we observed that diabetes increased the risk of a diagnosis of late stage breast cancer. This was constantly observed in the sensitivity analyses when only stage IV breast cancer was considered as late stage, or when articles with cancers in situ (or articles without distant metastasis) were excluded. Moreover, we confirmed the association between diabetes and lymph node invasion with adjusted estimates.

### 4.2. Tumor Stage, Tumor Size, Lymph Node Status, and Tumor Grade

The breast cancer stage at diagnosis determines treatments and prognosis. The TNM staging is the most widely used system [9], as well as in our included studies. To take full advantage of eligible studies, we pooled ORs that were accessed based on different staging systems (i.e., ORs of stages III/IV versus cancers in situ/stages I/II were combined with ORs of distant metastases/regional tumors versus localized tumors). Our finding showed that women with T2DM were predisposed to a later stage of breast cancer at diagnosis, and this was constantly confirmed in sensitivity analyses. Evidence that we obtained from the studies published in the last decade confirmed the results described in the past systematic review by Peairs et al. on the same topic in 2011 [29]. Moreover, we also observed that two important components of stage: tumor size and lymph node status, were significantly associated with T2DM, which further suggests an impact of T2DM on the progress of breast cancer. Our finding for lymph node status is in accordance with the results of a recent meta-analysis, although only crude risk ratios were pooled in that study [30], while only one article [62] was included in our meta-analysis. Sensitivity analyses showed that the associations between T2DM and tumor size and lymph node status became insignificant when the cutoffs were altered. This is likely in part due to the pooled small sample sizes. When leave-one-out sensitivity analysis was conducted for tumor grade, exclusion of one article [65] strengthened the association between diabetes and a high-grade tumor. That study, however, had a small sample size, and it included only patients using metformin. Metformin, which has been a first-line therapy for T2DM for half a century, is suspected to have anticancer effect [68,69], and the biologically plausible mechanisms could be activation of AMP-activated protein kinase, and then inhibition of mammalian target of rapamycin, a downstream effector of growth factor signaling [70]. Due to limited information, we could not explore the role of antidiabetic medication use in the relation between diabetes and breast tumor grade. 

The association between T2DM and late-stage breast tumor was supported by both epidemiological and biological evidence. Based on epidemiological articles, patients with diabetes are more likely to have a lower SES [71] and unhealthy habits [72,73], which are related to a lower participation rate in breast cancer screening programs [26]. As cancer screening decreases the incidence of late-stage breast cancer [15], a delayed detection could result in an aggressive tumor. From biological evidence in vitro, hyperglycemia in diabetes potentially creates a fertile ground for tumor growth, due to reliance on aerobic glycolysis (known as the Warburg effect) and therefore increased glucose consumption in cancer cells [74,75]. More importantly, hyperinsulinemia could stimulate carcinogenesis-related pathways to cause cancer cell proliferation, survival, and migration through insulin-like growth factors [76]. Hormonal changes in diabetes, such as increased bioavailable estrogen, could stimulate the proliferation of ER-positive and/or estrogen-dependent breast cancer [76]. Therefore, diabetes creates a favorable environment for accelerating cancer development and increase the likelihood of a late-stage breast cancer.

### 4.3. ER, PR and Her2 Status

To our knowledge, this is the first systematic review investigating the associations between diabetes and these three critical molecular biomarkers: ER, PR, and Her2. Although all the pORs failed to meet the statistical significance, we observed positive associations between T2DM and ER/PR expression in the studies [45,56], which were reverse to the overall trend of pORs. Since only patients with metformin or antidiabetic treatment were recruited in these studies, this observation alluded to a possibility that the associations between T2DM and hormone receptors could be a balance of influence from both diabetes and antidiabetic treatments. Based on available biological evidence, hyperglycemia or advanced glycation end products could upregulate ER expression [77,78]; on the other hand, metformin was capable of repressing the expression and transcriptional activity of ER and E2/ER-regulated genes (including PR) [79]. A recently published epidemiological article also supported that, compared with not having T2DM, T2DM with metformin use was associated with decreased risk of ER-positive breast cancer, but with increased risk of ER-negative breast cancer [80]. With regard to Her2, metformin could possibly play a role in downregulating Her2 expression [81], but less biological evidence is available to indicate the impact of diabetes on Her2 expression. Stratified analyses showed a significant inverse relationship between T2DM and Her2 expression in studies recruiting populations with a median calendar year of breast cancer diagnosis ≤2008, which might be intertwined by different Her2 testings with error rates [82]. Considering the borderline associations and limited sample sizes in our meta-analyses, the conclusions on the association between diabetes and these three biomarkers should be interpreted with caution.

### 4.4. Confounding Effect

Age and BMI are two shared risk factors between diabetes and breast cancer [83,84], and were considered as critical confounders in this current review. Age was adjusted or matched in all but one of the included studies. Since specific morphologic and prognostic characteristics of young-onset breast cancer were advocated in previous findings [38], such as enhanced likelihood to be a large and poorly differentiated tumor, or less often containing hormone receptors at diagnosis, we further stratified the studies by age at breast cancer diagnosis, and found an increased risk of tumors without PR expression in young patients. High BMI (≥25 kg/m^2^) was confirmed as another risk factor for developing breast cancer [85], and more specifically, obesity increases the risk for hormone receptor-positive breast cancer in postmenopausal women, but tends to increase the risk for TNBC in premenopausal women [86]. Evidence of the relation between BMI and Her2 status is limited [86]. Here, stratified analyses found a positive association between T2DM and lack of Her2 expression in studies [42,43] with BMI adjustment; these two studies had small sample sizes and recruited younger participants. These indicate the important confounding effect of age and BMI on the association between diabetes and tumor characteristics. However, limitations of studies and sample sizes constrained further exploration of these two factors.

Other than these two confounders, low SES is relevant to severity of diabetes [87], and a low screening rate and then aggressive tumors (e.g., less common to be hormone-receptor-positive tumors) [17,18]. A significant association between PR expression and diabetes was seen in the subgroup with two articles adjusting for SES [56,58]. Albeit limited sample sizes, this indicates a possible confounding role of SES in the relation between T2DM and tumor characteristics.

### 4.5. Strengths and Limitations

This systematic review and meta-analysis has two strengths. First, to our knowledge, this is the first meta-analysis based on epidemiological evidence to quantitatively evaluate the association between diabetic status and tumor stage, as well as tumor size; tumor grade; and ER, PR, and Her2 status in women diagnosed with breast cancer. Second, shared risk factors that could bias the association between diabetes and breast cancer were considered. We only pooled estimates adjusted for relevant confounders (or calculated estimates in articles with a paired design) in the meta-analysis, for the purpose to reduce the residual confounding and retrieve the best available evidence to our research question.

Our review also has some limitations: (1) most of included studies were rated low or median quality based on QUIPS. This was mostly due to insufficient description of patient selection or absent explanation of drop outs and missing values. The effect estimates of the relation between diabetes and tumor stage, and lymph node status were stronger in the subgroup of articles with overall high quality; (2) most of the included articles were cross-sectional studies, which often suffer the reversal causality problem [88]. During study selection, we excluded those determining diabetes after the diagnosis of breast cancer. Among the included studies, there are two studies with relatively small sample sizes [43,45] explicitly declaring a time window of 12 months between the diagnosis of diabetes and breast cancer. Each of them did not support a positive relation between diabetes and late-tumor stage. However, two included studies with large sample sizes [59,62] conducted stratified analyses by duration of diabetes, and both found a significant association between diabetes and advanced tumor stage. These findings highlight an important role of diabetes duration in the development of breast tumors, which should be considered in future studies; (3) ORs with adjustment for different confounders were pooled. Due to the limited number of studies, combining ORs with the exactly same confounders was infeasible. Since residual confounding in observational studies can never be excluded because of data availability and unknown characteristics [47], to reduce the possibility of over-estimation, only the ORs adjusted for relevant confounders were extracted and pooled; (4) the impact of T1DM on tumor characteristics could not be totally excluded. In included studies, four studies did not clearly mention whether they excluded T1DM or not, and two did mention they included a small portion of T1DM patients (Table S1; the numbers of T1DM and all the patients with DM: 25/211, 13/109). Considering the small proportion of T1DM, and elderly populations in the majority of studies, the impact of other diabetes seems to be not important.

### 4.6. Future Research Directions

As a supplement to elevated incidence risk and mortality risk of breast cancer in patients with diabetes, our findings offer further understanding of associations between these two diseases from a perspective of tumor characteristics. These results may contribute to the identification of women at high risk of aggressive breast cancers, and to the formulation of preventive measures and screening strategies. However, as aforementioned, our pooled estimates may be challenged by limitations, such as patient selection bias, reversal causality, insufficient adjustment of confounders, and misclassification of diabetes subtypes. Since diabetes is a group of metabolic disorders, the associations between T2DM and breast cancer characteristics could also be influenced by the metabolic status in patients [89]. Therefore, in the future, additional well-designed cohort studies are warranted with (1) explicit description of participants recruitment; (2) representative study populations, avoiding possible T2DM misclassification; (3) adequate control for confounding (e.g., adjustment for age and BMI); (4) taking into account the antidiabetic medication and the time window between the diagnosis of diabetes and the diagnosis of breast cancer; and (5) detailing the metabolic status (e.g., hyperglycemia and hyperinsulinemia) and hormonal changes in patients with T2DM.

## 5. Conclusions

This systematic review and meta-analysis provides evidence that pre-existing T2DM is associated with increased risk of late tumor stage, large tumor size, and invasive lymph node at the time of breast cancer diagnosis. Although many studies suffered from methodological limitations, the results were confirmed in sensitivity analyses in studies with a high quality. Given the high breast cancer risk and increased risk of poor breast cancers at diagnosis, we recommend that researchers further explore the role of breast screening in women with diabetes, and we urge physicians to be aware of enhanced risk of late-stage cancers, larger tumor sizes, and more lymph node invasion in women with diabetes.

## Figures and Tables

**Table 1:** Characteristics and results of studies included in the systematic review and meta-analysis.

First Author (Publication Year)	Study Design	Calendar Year of BC Diagnosis (Region)	Sample Size and Age (Years)		Original Comparisons: OR, 95% CI	Adjusted Factors ^6^	Matched Factors ^6^
			Breast Cancer with DM	Breast Cancer without DM			
Fahim (2021) [55]	CS	2007~2013 (USA)	sample size: 1719; age ^1^: <55, 55–64, 65–74, 75–84, >85: 45, 153, 657, 643, 221.	sample size: 6084; age ^1^: <55, 55–64, 65–74, 75–84, >85: 193, 328, 2238, 2172, 1153.	stages III/IV vs. cancers in situ and stages I/II: 0.97 (0.83–1.14).	age, race, geographic location, metropolitan status, comorbidity index, marital status, and year of BC diagnosis.	
Overbeek (2019) [56]	nested CC	2002~2014 (the Netherlands)	sample size: 1567; age: mean ± SD: 71 ± 11; ≤53/>53 ^1^: 85/1482.	sample size: 6267; age: mean ± SD: 71 ± 11; ≤53/>53 ^1^: 330/5937.	more advanced tumor stage: 1.28 (1.13–1.44); larger tumor size: 1.22 (1.08–1.38); more advanced lymph node status: 1.31 (1.12–1.53); more advanced grade: 1.22 (1.08,1.39); ER− vs. ER+: 1.04 (0.86–1.25); PR− vs. PR+: 0.77 (0.67–0.89); Her2+ vs. Her2-: 0.93 (0.75–1.16); more aggressive subtype: 1.10 (0.94–1.27).	age, year of BC diagnosis, SES, chronic disease score, and use of glucocorticoids, estrogen-progestogen contraceptives, and hormone replacement therapy in the year prior to the BC diagnosis.	age at BC diagnosis.
Chen (2019) [57]	CC	2004~2015; 2004~2012 (USA)	sample size: ER+/HER2-: 129; ER+/HER2+: 11; TN: 124; H2E: 46.	sample size: ER+/HER2-: 1863; ER+/HER2+: 313; TN: 1322; H2E: 532.	ER+/Her2+ vs. ER+/Her2-: 0.77 (0.40, 1.48); TNBC vs. ER+/Her2-: 1.38 (1.10, 1.89); H2E vs. ER+/Her2-: 1.38 (0.93, 2.06)	study site, year of BC diagnosis, BMI, age of BC diagnosis and race/ethnicity.	
			Age ^1^: <40, 40–49, 50–59, 60–69: ER+/Her2-: 268, 555, 639, 530; ER+/Her2+: 74, 119, 88, 43; TN: 206, 409, 457, 374; H2E: 70, 133, 220, 155.				
Ghanem (2019) [58]	CS	2016~2017 (Egypt)	sample size: 20; age: mean ± SD: 59.67 ± 4.03.	sample size: 20; age: mean ± SD: 57.27 ± 4.48	stage III vs. stages I/II: 2.45 (0.64–9.39); tumor size: >50 mm vs. ≤50 mm: 1.23 (0.35–4.31); lymph node invasion: N1–3 vs. N0: 2.45 (0.64–9.39); grade 3 vs. grade 1 and 2: 2.45 (0.64–9.39); grades 2 and 3 vs. grade 1: 1.89 (0.38–9.27); ER− vs. ER+: 0.46 (0.11–1.94); PR− vs. PR+: 0.66 (0.18–2.35).		menstrual state and SES.
Murto (2018) [59]	CS	1995~2013 (Finland)	sample size: 5469 age ^1^: ≤39, 40–55, ≥56:5, 207, 5257.	sample size: 67,701 age ^1^: ≤39, 40–55, ≥56: 570, 8311, 58, 820.	tumor stage: locally advanced vs. localized: 1.26 (1.18, 1.35); distant metastases vs. localized: 1.59 (1.44, 1.75).	age, number of mammography, screening rounds attended before the BC diagnosis, hypercholesterolemia, hypertension, coronary artery disease and obesity.	
Alsaeed (2017) [42]	CS	2000~2006 (Saudi)	sample size: 24;	sample size: 86;	ER+ vs. ER−: 0.72 (0.22, 2.32); Her2+ vs. Her2-: 0.40 (0.12, 1.13).	obesity, hypertension and dyslipidemia.	
			Age ^1^:<25, 25–35, 36–45, >45: 2, 13, 29, 66.				
Mu (2017) [60]	CS	2005~2010 (China)	sample size: 462; age ^1^: <50, ≥50-<65, ≥65: 61, 256, 145.	sample size: 1644; age ^1^:<50, ≥50-<65, ≥65:237, 896, 511.	tumor size: >20 mm vs. ≤20 mm: 1.09 (0.85–1.40) ;>50 mm vs. ≤50 mm: 1.47 (1.07–2.02); lymph node status: N1–3 vs. N0: 1.04 (0.85–1.28); grades 2 and 3 vs. grade 1: 1.45 (1.08–1.94); ER− vs. ER+: 1.177 (0.96–1.45); PR− vs. PR+: 1.06 (0.86–1.31); Her2+ vs. Her2-: 1.13 (0.90–1.42).		the same recruiting period and matched with age.
Bronsveld (2016) [43]	CS	2000~2010 (Denmark)	sample size: 211; age: median, IQR in two strata of age (≤50, >50): 47.0 (43.0, 50.0); 67.0 (60.0, 75.0).	sample size: 101; age: median, IQR in two strata of age (≤50, >50): 47.0 (43.0, 50.0); 67.0 (62.0, 73.0).	tumor size ^2^: >20 mm vs. ≤20 mm: 0.98 (0.61–1.59); >50 mm vs. ≤50 mm: 1.97 (0.54–7.14); lymph node status ^2^: N1–3 vs. N0: 1.16 (0.72–1.88); N2/N3 vs. N0/N1: 0.83 (0.45–1.53); grade 2 vs. grade 1 (pre-, post-menopausal):0.56 (0.22,1.42), 0.80 (0.31,2.03); grade 3 vs. grade 1 (pre-, post-menopausal): 1.08 (0.41, 2.86), 1.97 (0.72, 5.39); ER− vs. ER+ (pre-, post-menopausal): 2.32 (0.86, 6.31), 1.33 (0.52, 3.40); PR− vs. PR+ (pre-, post-menopausal):2.18 (0.92, 5.17), 1.06 (0.51, 2.19); Her2- vs. Her2+ (pre-, post-menopausal) :2.94 (1.08, 8.02), 1.20 (0.40, 3.59); luminal B-like, Her2- vs. luminal A-like (pre-, post-menopausal): 1.05 (0.40–2.73), 0.58 (0.25–1.35); Her2+ vs. luminal A-like (pre-, post-menopausal): 0.41 (0.14, 1.20), 0.88 (0.28, 2.71); TNBC vs. luminal A-like (pre-, post-menopausal): 2.21 (0.71, 6.69), 1.30 (0.40, 4.20).	age and BMI, except for grade, which was adjusted for age only.	year of birth and age at diagnosis (both in 10-year categories).
Samson (2016) [61]	CS	1993~2002; 1996~2001 (USA)	sample size: African-American: 170; European-American: 73.	sample size: African-American: 509; European-American: 619.	Stage ^3^: localized vs. in situ: 1.23 (0.33, 4.54); regional vs. in situ: 1.34 (0.36, 5.05); distant metastasis vs. in situ: 1.36 (0.22, 8.59).	diabetes medications, and menopausal (deduced by age).	
			age: mean ± SD: African-American: 61 ± 12; European-American: 63 ± 13.				
Lipscombe (2015) [62]	CS	2007~2012 (Canada)	sample size: 6115; age: median, IQR: 68 (60, 77).	sample size: 32,292; age: median, IQR: 59 (50, 69).	stage II vs. stage I: 1.14 (1.07, 1.22); stage III vs. stage I: 1.21 (1.11, 1.33); stage IV vs. stage I: 1.16 (1.01, 1.33); tumor size: > 20 mm vs. ≤20 mm: 1.16 (1.06, 1.28); lymph node status: N1–3 vs. N0: 1.16 (1.06–1.27); ER+ vs. ER−: 1.01 (0.93, 1.10).	prior screening mammogram, age, neighborhood income quintile, rural residence, number of primary care visits, weighted ADG comorbidity score, renal dialysis, and history of acute myocardial infarction, stroke, or congestive heart failure.	
Li (2015) [63]	CS	2009~2012 (China)	sample size: 98; age: mean ± SD: 57.3 ± 10.3.	sample size: 107; age: mean ± SD: 56.6 ± 11.1.	tumor size: >20 mm vs. ≤20 mm: 1.83 (1.03–3.23); lymph node status: N1–3 vs. N0: 1.85 (1.06–3.22); grade 3 vs. grade 1–2: 1.67 (0.81–3.47); ER− vs. ER+: 1.05 (0.59–1.87); PR− vs. PR+: 1.31 (0.75–2.29); Her2+ vs. Her2-: 1.40 (0.73–2.71);		the same admitting period and age.
Karlin (2014) [64]	CS	2007~2011 (USA)	sample size: 109; age: median, range: 68 (28–91).	sample size: 109; age: median, range: 68 (28–91).	stages III/IV vs. stages I/II: 1.011 (0.466–2.195); grades 2 and 3 vs. grade 1: 1.06 (0.54–2.11); grade 3 vs. grades 1 and 2: 1.41 (0.76–2.60); ER− vs. ER+: 0.978 (0.461–2.074); PR− vs. PR+: 0.86 (0.48–1.55); Her2+ vs. Her2-: 1.710 (0.737–3.971).		age at diagnosis of BC, race, ethnicity, and year of BC diagnosis.
Wang (2014) [44]	CS	2007~2013 (China)	sample size: 164; age: mean: 60.7.	sample size: the first control group (nondiabetic patients with breast cancer): 328; age: unknown.	tumor size ^4^: >20 mm vs. ≤20 mm: 0.82 (0.54, 1.23); >50 mm vs. ≤50 mm: 1.44 (0.78, 2.64); lymph node status ^4^: N1–3 vs. N0:1.69 (1.12–2.56); N2/N3 vs. N1/N2: 1.85 (1.14–3.01); grade 3 vs. grades 1 and 2 ^4^: 1.02 (0.46–2.27);grades 2 and 3 vs. grade 1 ^4^: 0.86 (0.42–1.79);ER− vs. ER+ ^4^: 1.26 (0.84–1.88); PR− vs. PR+ ^4^: 0.79 (0.54–1.17); Her2+ vs. Her2- ^4,5^: 1.10 (0.74–1.63).		age at the time of BC diagnosis (±5 years), and year of diagnosis (±5 years).
Aksoy (2013) [45]	CS	2000~2012 (Turkey)	sample size: 148; age ^1^: <50/≥50: 37/111.	sample size: 636; age ^1^: <50/≥50: 148/488.	stages III/IV vs. stages I/II: 1.15 (0.78–1.68);tumor size: >50 mm vs. ≤5 mm: 1.23 (0.77, 1.96); lymph node invasion: N1–3 vs. N0: 1.07 (0.72–1.59); N2/N3 vs. N0: 1.34 (0.89–2.03); grade 3 vs. grades 1 and 2: 0.61 (0.40–0.92); grades 2/3 vs. grade 1: 1.05 (0.61–1.80); ER− vs. ER+: 0.55 (0.34–0.89); PR− vs. PR+: 0.58 (0.37–0.91); Her2+ vs. Her2-: 0.77 (0.47–1.26); TNBC: yes vs. no: 0.41 (0.19–0.86).		age.
Ferro (2013) [65]	CS	2004~2012 (USA)	sample size: 51; age: mean: 60.02;>50/≤50 ^1^: 44/7.	sample size: 51; age: mean: 57.75; >50/≤50 ^1^: 39/12.	tumor size: >20 mm vs. ≤20 mm: 1.18 (0.50–2.76); >50 mm vs. ≤50 mm: 0.32 (0.06–1.67); grade 3 vs. grades 1 and 2: 1.38 (0.56–3.41); grades 2 and 3 vs. grade 1: 0.43 (0.10–1.80); ER− vs. ER+: 1.21 (0.50–2.94); PR− vs. PR+: 1.10 (0.47–2.56); Her2+ vs. Her2-: 0.32 (0.11–0.91);		age (±5 years), surgical procedure, presence of adjuvant chemotherapy, radiation field design, and radiation dose.
Yerrabothala (2013) [66]	CS	2001~2010 (USA)	sample size: 85; age: median, IQR: 61 (55, 71).	sample size: 170; age: median, IQR: 62 (55, 71).	stages III/IV vs. cancers in situ and stages I/II: 1.51 (0.81–2.80); ER− vs. ER+: 2.63 (1.36–5.10); PR− vs. PR+: 2.22 (1.30–3.79); Her2+ vs. Her2-: 1.47 (0.71–3.01); TNBC: yes vs. no: 3.69 (1.70–8.04).		age (±2 years).
Liao (2010) [67]	CS	2005~2009 (China)	sample size: 143; age: mean ± SD: 58.3 ± 10.0.	sample size: 143; age: mean ± SD: 49.1 ± 11.7.	tumor size: >50 mm vs. ≤50 mm: 1.42 (0.67, 2.98); lymph node status: N2/N3 vs. N0/N1: 16.32 (5.54, 48.0); ER− vs. ER+: 1.40 (0.77, 2.53); PR− vs. PR+: 2.10 (1.13, 3.89); Her2- vs. Her2+: 1.76 (0.98, 3.14).	age.	the same admitting period.

^1^ Numbers of patients in different age groups; ^2^ adjusted ORs were unavailable for tumor size and lymph node status, so we re-calculated the ORs since it was also a study with a matching study design; ^3^ only data from the African-American population were used, because one of the comparisons for European-American patients (distant metastasis versus in situ) was missing due to a small sample size, and the Hamling et al. [46] method could not be used to get expected comparisons; ^4^ just use the first control group as the reference group; ^5^ borderline of Her2 was treated as positive when calculating OR; ^6^ adjusted ORs were extracted whenever possible; otherwise, ORs were recalculated based on the frequency table in case of a matching study design. DM, diabetes mellitus; BC, breast cancer; SES, socioeconomic status; BMI, body mass index; SD, standard deviation; IQR, interquartile range; OR, odds ratio; CI, confidence interval; CS, cross-sectional; CC, case-control; ER, estrogen receptor; PR, progesterone receptor; Her2, human epidermal growth factor receptor-2; H2E, ER-/PR-/Her2+; TNBC, triple-negative breast cancer.

## Data Availability

Data is contained within the article or supplementary material.

## Supplementary Materials

The following are available online at https://www.mdpi.com/article/10.3390/cancers13194992/s1, Material S1: Search strategy, Material S2: QUIPS: Risk of Bias (ROB) Assessment Instrument for Prognostic Factor Studies, Material S3: Data transformation in case of different comparisons or estimates, Figure S1: Stratified analyses for finding possible interpretation for heterogeneity in the association between T2DM and tumor stage, Figure S2: Stratified analyses for finding possible interpretation for heterogeneity in the association between T2DM and lymph node status, Figure S3: Stratified analyses for finding possible interpretation for heterogeneity in the association between T2DM and ER status, Figure S4: Stratified analyses for finding possible interpretation for heterogeneity in the association between T2DM and PR status, Figure S5: Stratified analyses for finding possible interpretation for heterogeneity in the association between T2DM and Her2 expression, Figure S6: Funnel plots for T2DM and breast tumor characteristics, Table S1: Additional characteristics of studies included in the systematic review and meta-analysis, Table S2: Methodological quality of the included studies based on the QUIPS tool, Table S3: Sensitivity analyses.
