# Supplementary material for: Type 2 Diabetes Mellitus and Clinicopathological Tumor Characteristics in Women Diagnosed with Breast Cancer: A Systematic Review and Meta-Analysis
Source: Cancers (Basel). 2021 Oct 5;13(19):4992. doi: 10.3390/cancers13194992 (PMC8508341; doi:10.3390/cancers13194992)
Supplement: Supplementary file 1 [file cancers-13-04992-s001.zip › cancers-1343114-supplementary.pdf]

*Supplementary Materials*

# Type 2 Diabetes Mellitus and Clinicopathological Tumor Characteristics in Women Diagnosed with Breast Cancer: A Systematic Review and Meta-Analysis

**Fan Zhang, Jing de Haan-Du, Grigory Sidorenkov, Gijs W. D. Landman, Mathilde Jalving, Qingying Zhang and Geertruida H. de Bock**

This file contains supplementary materials S1, S2 and S3, six supplementary figures (Figure S1–S6), and three supplementary tables (Table S1–S3).

**Material S1. Search Strategy**

A systematic database search in Pubmed, Embase and Web of Science was conducted from January 1, 2010 to July 2, 2021. Search strings included MeSH terms and free text words on breast cancer, diabetes, and tumor features. To avoid missing qualified data in studies mainly examining the prognosis or the incidence of breast cancer in women with diabetes, keywords including survival, recurrence and risk were also included into the search strategy.

*1. Search Strategy for Pubmed*

("breast neoplasms"[MeSH] OR breast neoplas*[tiab] OR breast cance*[tiab] OR breast carcinom*[tiab] OR breast tumo*[tiab] OR breast adenocarcinom*[tiab])

AND

("diabetes mellitus"[MeSH] OR diabetes[tiab] OR diabetic*[tiab])

AND

("neoplasm staging"[MeSH] OR "lymph nodes"[MeSH] OR TNM[tiab] OR tumor siz*[tiab] OR tumour siz*[tiab] OR lymph nod*[tiab] OR stage*[tiab] OR staging[tiab] OR grade*[tiab] OR grading[tiab]

OR "receptors, progesterone"[MeSH] OR "receptors, estrogen"[MeSH] OR "ERBB2 protein, human" [Supplementary Concept] OR "ErbB Receptors"[Mesh] OR epidermal growth factor receptor 2[tiab] OR ERBB2[tiab] OR her2[tiab] OR progesterone receptor*[tiab] OR estrogen receptor*[tiab] OR oestrogen receptor*[tiab] OR hormone receptor*[tiab]

OR "prognosis"[MeSH] OR "survival"[MeSH] OR "mortality"[MeSH] OR "death"[MeSH] OR "recurrence"[MeSH] OR "incidence"[MeSH] OR "risk"[MeSH] OR survival*[tiab] OR mortalit*[tiab] OR death*[tiab] OR prognos*[tiab] OR recurren*[tiab] OR relapse*[tiab] OR metastas*[tiab] OR incidence*[tiab] OR risk*[tiab])

AND

("2010/01/01"[Date - Publication]: "2021/07/02"[Date - Publication])

NOT

("Animals"[Mesh] NOT "Humans"[Mesh])

*2. Search Strategy for Web of Science (Filter: from 2010 to 2021):*

(TS="diabetes" OR TS="diabetic*")

AND (TS="breast neoplas*" OR TS="breast cance*" OR TS="breast carcinom*" OR TS="breast tumo*" OR TS="breast adenocarcinom*")

AND (TS="neoplasm staging" OR TS="lymph nod*" OR TS=stage OR TS=stages OR TS=staging OR TS=TNM OR TS="tumor siz*" OR TS="tumour siz*" OR TS=grade* OR TS=grading

OR TS="epidermal growth factor receptor 2" OR TS=ERBB2 OR TS=her2 OR TS="progesterone receptor*" OR TS="estrogen receptor*" OR TS="oestrogen receptor*" OR TS="hormone receptor*"

OR TS=survival* OR TS=mortalit* OR TS=death* OR TS=prognos* OR TS=recurren* OR TS=relapse* OR TS=metastas* OR TS=incidence* OR TS=risk*)

*3. Search Strategy for Embase:*

| | |
|---|---|
| #1 | 'breast cancer'/exp OR 'breast tumor'/exp OR ('breast neoplas*' OR 'breast carcinom*' OR 'breast tumo*' OR 'breast adenocarcinom*'):ab,ti |
| #2 | 'diabetes mellitus'/exp OR (diabetes OR diabetic*):ab,ti |
| #3 | 'cancer diagnosis'/exp OR 'cancer staging'/exp OR 'hormone receptor'/exp OR 'prognosis'/exp OR 'survival'/exp OR 'mortality'/exp OR 'death'/exp OR 'incidence'/exp OR 'risk'/exp OR 'recurrent disease'/exp OR (stage* OR staging OR TNM OR tumor siz* OR tumour siz* OR lymph nod* OR grade* OR grading OR epidermal growth factor receptor 2 OR ERBB2 OR her2 OR progesterone receptor* OR estrogen receptor* OR oestrogen receptor* OR hormone receptor* OR survival* OR mortalit* OR death* OR prognos* OR recurren* OR relapse* OR metastas* OR incidence* OR risk*):ab,ti |
| #4 | #1 AND #2 AND #3 AND [2010-2021]/py NOT ([animals]/lim NOT [humans]/lim) NOT 'conference abstract'/it |

**Material S2. QUIPS: Risk of Bias (ROB) Assessment Instrument for Prognostic Factor Studies**

| **Domain** | **Issues to consider for judging overall rating of ROB** |
|---|---|
| **1. Study Participation** | **Goal: To judge the risk of selection bias.**<br>**(likelihood that relationship between diabetes and tumor characteristics is different for participants and eligible non-participants).** |
| *Source of target population* | There is adequately description about where to recruit participants (e.g., registration or database, or medical records) and why to recruit participants (e.g., detection of diabetic effect on prognosis, or detection of association between diabetes and breast cancer features). |
| *Method used to identify population* | There is adequately description about sample frame (e.g., consecutive samples, or selective samples with clear description of randomly selection), and participation rate (the proportion of included samples in the eligible population). |
| *Recruitment period* | Period of recruitment is adequately described. |
| *Place of recruitment* | Place of recruitment (setting and geographic location) are adequately described. |
| *Inclusion and exclusion criteria* | There is an adequately description in text (or a flow chart) with numbers of samples in each step, and no restriction on the pre-specified tumor features. |
| *Adequate study participation* | There is adequate participation in the study by eligible individuals. |
| *Baseline characteristics* | There is key characteristics (at least age and BMI) with acceptable missing rates, and they explained the reason why there were missing. |
| **Summary Study participation** | **The study sample represents the population of interest on key characteristics, sufficient to limit potential bias of the observed relationship between diabetes and tumor characteristics.** |
| | |
| **2. Study Attrition** | **Goal: To judge the risk of attrition bias, and as for case-control and cross-sectional studies, this domain is not applicable.**<br>**(likelihood that relationship between diabetes and tumor characteristics are different for completing and non-completing participants).** |
| *Proportion of baseline sample available for analysis* | There is acceptable missing rate of tumor stage (the primary outcome) or other secondary outcomes. |
| *Attempts to collect information on participants who dropped out* | Comprehensively consider the missing rate and attempt to collect information. |
| *Reasons and potential impact of subjects lost to follow-up* | Comprehensively consider the missing rate and provide reasons for loss to follow-up. |
| | There is adequately description on key characteristics: at least for age and BMI, for participants lost to follow up. |

| | |
|---|---|
| *Outcome and prognostic factor information on those lost to follow-up* | There is no important difference between key characteristics and outcomes in participants who completed the study and those who did not. |
| **Study Attrition Summary** | **Loss to follow-up (from baseline sample to study population analyzed) is not associated with key characteristics (i.e., the study data adequately represent the sample) sufficient to limit potential bias to the observed relationship between diabetes and tumor characteristics.** |
| | |
| **3. Prognostic Factor Measurement** | **Goal: To judge the risk of measurement bias related to how diabetes was measured.**<br>**(differential measurement of diabetes related to the level of tumor characteristics).** |
| *Definition of the PF* | There is a clear description of diabetes to reduce the possibility of misclassification. |
| *Valid and Reliable Measurement of PF* | Medical record and HbA1c test results are regarded as reliable measurement of diabetes, and then is medical claims (insurance), and self-reported diagnosis/medication use/once fast glucose measurement. |
| *Method and Setting of PF Measurement* | The same method of measurement was used for all study participants. |
| *Proportion of data on PF available for analysis* | There is adequate proportion of the study samples with complete information for diabetes. |
| *Method used for missing data* | Appropriate methods of imputation were used for missing diabetic status for samples. |
| **PF Measurement Summary** | **Diabetes is adequately measured in study participants to sufficiently limit potential bias.** |
| | |
| **4. Outcome Measurement** | **Goal: To judge ROB related to the measurement of tumor characteristics.**<br>**(differential measurement of outcome related to the baseline diabetic status).** |
| *Definition of the Outcome* | A clear definition of tumor stage (or secondary outcomes) is provided, or information is extracted from well-organized databases or medical records. |
| *Valid and Reliable Measurement of Outcome* | Medical record or well-organized database is reliable. |
| *Method and Setting of Outcome Measurement* | The same measurement of outcome was used for all participants. |
| **Outcome Measurement Summary** | **Outcome of interest is adequately measured in study participants to sufficiently limit potential bias.** |
| | |
| **5. Study Confounding** | **Goal: To judge the risk of bias due to confounding.**<br>**(i.e., the effect of diabetes is distorted by another factor that is related to diabetes and tumor characteristics).** |
| *Important Confounders Measured* | Key variables (at least age and BMI) were measured. |

| | |
|---|---|
| *Definition of the confounding factor* | Clear definitions of the confounding factors are provided, or information is extracted from well-organized databases or medical records. |
| *Valid and Reliable Measurement of Confounders* | Medical record or well-organized database is reliable. |
| *Method and Setting of Confounding Measurement* | The same source of confounding information was used for all participants |
| *Method used for missing data* | Appropriate methods are used to impute missing values for confounders or treat missing values as another category. |
| *Appropriate Accounting for Confounding* | Samples were matched for key variables (e.g., age and BMI) in the study design. |
| | Adjustment according to important confounders (i.e., age and BMI) was used. |
| **Study Confounding Summary** | **Important potential confounders are appropriately accounted for, limiting potential bias with respect to the relationship between diabetes and *tumor characteristics*.** |
| | |
| **6. Statistical Analysis and Reporting** | **Goal: To judge ROB related to the statistical analysis and presentation of results.** |
| *Presentation of analytical strategy* | There is a large enough sample size considering the statistical model (there is a minimum of 10 patients per covariate in the smallest group). |
| *Model development strategy* | Analyses control the most important factors (e.g., age and BMI), and any additional factors. |
| | The selected statistical model is appropriate for the design of the study. |
| *Reporting of results* | There is no selective reporting of results. |
| **Statistical Analysis and Presentation Summary** | **The statistical analysis is appropriate for the design of the study, limiting potential for presentation of invalid or spurious results.** |

**Material S3: Data transformation in case of different comparisons or estimates**

1. In case that the association represents "non-diabetes and increased risk of aggressive tumor types", or "diabetes and decreased risk to get disadvantageous tumor types at diagnosis", OR (odds ratio) was recalculated by 1/OR, and the 95% CI is calculated likewise;

2. Whenever possible, we directly extracted adjusted ORs of expected comparisons from individual studies (e.g. ORs for stage III-IV vs. cancers and stage I-II); however, in several studies, only one category (e.g. stage I) fell in the reference group while the other categories were treated as different levels of outcomes (e.g. stage II, stage III and stage IV). In this case, we utilized the method proposed by Hamling et al. [1] to firstly estimate the effective numbers of subjects in each category representing the 'adjusted' population and then to obtain OR for the expected comparison within this particular study;

3. One included study [2] provided adjusted ORs separately for pre- and post-menopausal patients; in this case, a fixed effect model was applied to compute a representative pooled OR for this study.

**Data transformation in case of different comparisons.**

| Outcomes | Author, year | Original comparisons: OR, 95% CI | Transformed effect sizes: OR, 95% CI |
|---|---|---|---|
| Tumor stage | Murto, 2018 | locally advanced vs. localized: 1.26 (1.18, 1.35);<br>distant metastases vs. localized: 1.59 (1.44, 1.75). | distant metastases/locally advanced vs. localized [1]: 1.33 (1.25, 1.42); |
| | Samson, 2016 | African-American:<br>localized vs. in situ: 1.23 (0.33, 4.54);<br>regional vs. in situ: 1.34 (0.36, 5.05);<br>distant vs. in situ: 1.36 (0.22, 8.59). | African-American:<br>distant/regional vs. localized/in situ [1]:<br>1.14 (0.52, 2.51) |
| | Lipscombe, 2015 | stage 2 vs. stage 1: 1.14 (1.07, 1.22);<br>stage 3 vs. stage 1: 1.21 (1.11, 1.33);<br>stage 4 vs. stage 1: 1.16 (1.01, 1.33); | stage 3–4 vs. stage 1–2 [1]:<br>1.12 (1.04, 1.21) |
| Tumor grade | Bronsveld, 2016 | Premenopausal:<br>grade 2 vs. grade 1: 0.56 (0.22,1.42);<br>grade 3 vs. grade 1: 1.08 (0.41, 2.86);<br>Postemenopausal:<br>grade 2 vs. grade 1: 0.80 (0.31,2.03);<br>grade 3 vs. grade 1: 1.97 (0.72, 5.39). | grade 2–3 vs. grade 1 [1, 2]:<br>0.94 (0.51, 1.75).<br><br>grade 3 vs. grade 1–2 [1, 2]:<br>1.90 (1.13, 3.21). |
| ER expression | Alsaeed, 2017 | ER+ vs. ER-: 0.72 (0.22, 2.32) | ER- vs. ER+ [3]: 1.39 (0.43, 4.55) |
| | Bronsveld, 2016 | Premenopausal: ER- vs. ER+: 2.32 (0.86, 6.31);<br>Postmenopausal: ER- vs. ER+: 1.33 (0.52, 3.40). | ER- vs. ER+ [2]: 1.73 (0.87, 3.42) |
| | Lipscombe, 2015 | ER+ vs. ER-: 1.01 (0.93, 1.10). | ER- vs. ER+ [3]: 0.99 (0.91, 1.08) |
| PR expression | Alsaeed, 2017 | PR+ vs. PR-: 0.66 (0.20, 2.18) | PR- vs. PR+ [3]: 1.52 (0.46, 5.00) |
| | Bronsveld, 2016 | Premenopausal: PR- vs. PR+: 2.18 (0.92, 5.17);<br>Postmenopausal: PR- vs. PR+: 1.06 (0.51, 2.19). | PR- vs. PR+ [2]: 1.43 (0.82, 2.50) |
| Her2 expression | Bronsveld, 2016 | Premenopausal: Her2+ vs. Her2-: 0.34 (0.12, 0.93);<br>Postmenopausal: Her2+ vs. Her2-: 0.83 (0.28, 2.50) | Her2+ vs. Her2- [2]:0.52 (0.24, 1.09) |
| | Liao, 2010 | Her2- vs. Her2+: 1.76 (0.98, 3.14) | Her2+ vs. Her2- [3]: 0.57 (0.32, 1.02) |
| TNBC | Chen, 2019 | ER+/Her2+ vs. ER+/Her2-: 0.77 (0.40, 1.48);<br>TNBC vs. ER+/Her2-: 1.38 (1.10, 1.89);<br>H2E vs. ER+/Her2-: 1.38 (0.93, 2.06). | TNBC vs. non-TNBC [1]: 1.32 (1.03, 1.69) |
| | Bronsveld, 2016 | Premenopausal:<br>luminal B-like, Her2- vs. luminal A-like: 1.05 (0.40-2.73);<br>Her2+ vs. luminal A-like: 0.41 (0.14, 1.20);<br>TNBC vs. luminal A-like: 2.21 (0.71, 6.69).<br>Postmenopausal:<br>luminal B-like, Her2- vs. luminal A-like: 0.58 (0.25-1.35);<br>Her2+ vs. luminal A-like: 0.88 (0.28, 2.71);<br>TNBC vs. luminal A-like: 1.30 (0.40, 4.20). | TNBC vs. non-TNBC [1, 2]:<br>2.03 (0.94, 4.39) |

[1] use Hamling et al. [1] method to obtain OR for the expected comparison within the particular study; [2] a fixed effect model was applied to compute a pooled OR for pre- and post-menopausal effect sizes within the study [2]; [3] OR was recalculated by 1/OR, and the 95% CI is calculated likewise. OR: odds ratio; CI, confidence interval; ER: estrogen receptor; PR: progesterone receptor; Her2: human epidermal growth factor receptor-2; TNBC: triple-negative breast cancer; H2E: ER-/PR-/Her2+.

**Tumor stage**

| Study characteristics | N | Sample sizes T2DM | Sample sizes non-T2DM | pORs | 95% CI | I-squared | P_Qtest |
|---|---|---|---|---|---|---|---|
| **Region** | | | | | | | |
| eatern | 2 | 155 | 596 | 1.34 | 0.69, 2.60 | 0.12 | 0.286 |
| western | 7 | 14632 | 101533 | 1.18 | 1.06, 1.32 | 0.72 | 0.002 |
| **Median calendar year of BC diagnosis** | | | | | | | |
| >2008 | 4 | 7956 | 38499 | 1.08 | 0.79, 1.48 | 0.25 | 0.260 |
| ≤2008 | 5 | 6831 | 63630 | 1.31 | 1.20, 1.42 | 0.00 | 0.895 |
| **Sample size** | | | | | | | |
| >1000 | 4 | 14275 | 100751 | 1.18 | 1.03, 1.35 | 0.85 | <0.001 |
| ≤1000 | 5 | 512 | 1378 | 1.25 | 0.89, 1.74 | 0.00 | 0.764 |
| **SES** | | | | | | | |
| adjusted | 3 | 7695 | 38528 | 1.24 | 0.90, 1.70 | 0.56 | 0.103 |
| not adjusted | 6 | 7092 | 63601 | 1.17 | 1.01, 1.36 | 0.64 | 0.015 |
| **Comorbidity score** | | | | | | | |
| adjusted | 3 | 9394 | 44592 | 1.12 | 0.97, 1.30 | 0.74 | 0.022 |
| not adjusted | 6 | 5393 | 57537 | 1.29 | 1.04, 1.60 | 0.00 | 0.830 |
| **Screening considered** | | | | | | | |
| no | 7 | 3791 | 13678 | 1.17 | 0.96, 1.42 | 0.36 | 0.150 |
| yes | 2 | 10996 | 88451 | 1.22 | 1.04, 1.44 | 0.91 | 0.001 |
| **Overall quality** | | | | | | | |
| high | 2 | 6441 | 62375 | 1.32 | 1.24, 1.40 | 0.00 | 0.583 |
| low/median | 7 | 8346 | 39754 | 1.12 | 0.92, 1.36 | 0.00 | 0.529 |
| **ROB of study participation** | | | | | | | |
| low | 2 | 6441 | 62375 | 1.32 | 1.24, 1.40 | 0.00 | 0.583 |
| median/high | 7 | 8346 | 39754 | 1.12 | 0.92, 1.36 | 0.00 | 0.529 |
| **Staging system** | | | | | | | |
| staging system of invasive tumors | 2 | 5051 | 56668 | 1.33 | 1.22, 1.44 | 0.00 | 0.702 |
| TNM staging system | 7 | 9736 | 45461 | 1.15 | 0.97, 1.38 | 0.39 | 0.132 |

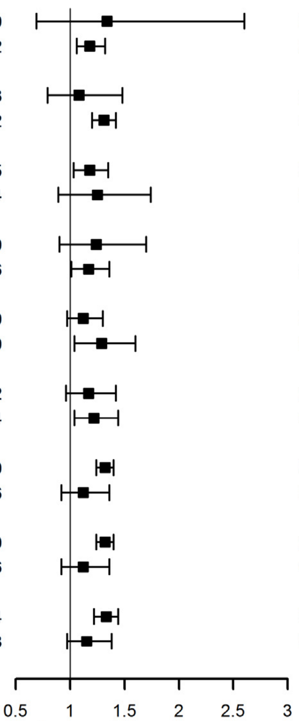


**Figure S1.** Stratified analyses to find possible interpretation for heterogeneity in the association between T2DM and tumor stage. Staging system of invasive tumor categories: localized, regional/locally advanced tumors, and distant metastases; N: the number of studies; T2DM: type 2 diabetes mellitus; BC: breast cancer; SES: socioeconomic status; pORs: pooled odds ratios; CI, confidence interval; ROB: risk of bias; P_Qtest: *P*-value from Q-test.

**Lymph node status**

| Study characteristics | N | Sample T2DM | sizes non-T2DM | pORs | 95% CI | I-squared | P_Qtest |
|---|---|---|---|---|---|---|---|
| **Region** | | | | | | | |
| eatern | 5 | 845 | 2557 | 1.34 | 0.98, 1.83 | 0.52 | 0.083 |
| western | 3 | 4357 | 19542 | 1.20 | 1.09, 1.33 | 0.00 | 0.414 |
| **Median calendar year of BC diagnosis** | | | | | | | |
| >2008 | 4 | 2899 | 13897 | 1.44 | 1.07, 1.94 | 0.55 | 0.086 |
| ≤2008 | 4 | 2303 | 8202 | 1.17 | 1.02, 1.35 | 0.12 | 0.333 |
| **Sample size** | | | | | | | |
| >1000 | 3 | 4616 | 21086 | 1.17 | 1.04, 1.33 | 0.40 | 0.191 |
| ≤1000 | 5 | 586 | 1013 | 1.42 | 1.05, 1.92 | 0.17 | 0.309 |
| **Proportion of young participants** | | | | | | | |
| >25 | 2 | 301 | 207 | 1.44 | 0.89, 2.31 | 0.36 | 0.213 |
| ≤25 | 4 | 4742 | 21594 | 1.17 | 1.05, 1.30 | 0.15 | 0.318 |
| unknown | 2 | 159 | 298 | 1.75 | 1.14, 2.69 | 0.00 | 0.600 |
| **SES** | | | | | | | |
| adjusted | 3 | 4174 | 19462 | 1.27 | 0.94, 1.71 | 0.30 | 0.240 |
| not adjusted | 5 | 1028 | 2637 | 1.26 | 0.98, 1.62 | 0.44 | 0.130 |
| **Comorbidity score** | | | | | | | |
| adjusted | 2 | 4154 | 19442 | 1.21 | 1.08, 1.36 | 0.43 | 0.186 |
| not adjusted | 6 | 1048 | 2657 | 1.30 | 1.00, 1.70 | 0.39 | 0.142 |
| **Time window ≥1 year** | | | | | | | |
| no | 6 | 4873 | 21491 | 1.31 | 1.05, 1.63 | 0.48 | 0.084 |
| yes | 2 | 329 | 608 | 1.11 | 0.81, 1.50 | 0.00 | 0.799 |
| **Overall quality** | | | | | | | |
| high | 2 | 1715 | 6050 | 1.29 | 1.11, 1.51 | 0.00 | 0.637 |
| low/median | 6 | 3487 | 16049 | 1.28 | 1.00, 1.64 | 0.40 | 0.139 |
| **ROB of study participation** | | | | | | | |
| low | 2 | 1715 | 6050 | 1.29 | 1.11, 1.51 | 0.00 | 0.637 |
| median/high | 6 | 3487 | 16049 | 1.28 | 1.00, 1.64 | 0.40 | 0.139 |

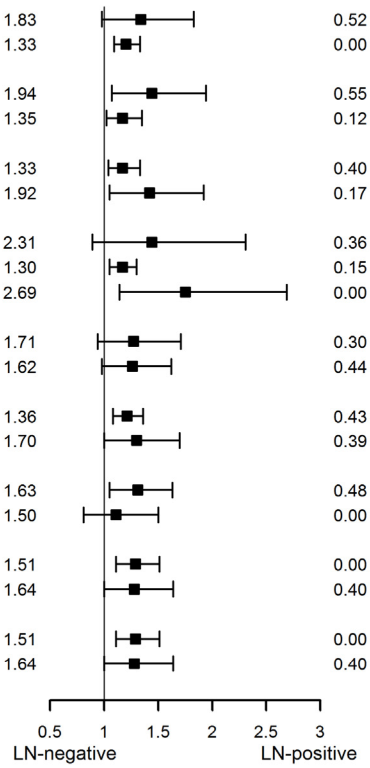


**Figure S2.** Stratified analyses to find possible interpretation for heterogeneity in the association between T2DM and lymph node status. N: the number of studies; T2DM: type 2 diabetes mellitus; BC: breast cancer; LN: lymph node; SES: socioeconomic status; pORs: pooled odds ratios; CI, confidence interval; ROB: risk of bias; P_Qtest: *P*-value from Q-test.

**ER status**

| Study characteristics | N | Sample sizes T2DM | Sample sizes non-T2DM | pORs | 95% CI | I-squared | P_Qtest |
|---|---|---|---|---|---|---|---|
| **Region** | | | | | | | |
| eatern | 7 | 991 | 2854 | 1.02 | 0.74, 1.42 | 0.46 | 0.086 |
| western | 6 | 5161 | 21877 | 1.24 | 0.90, 1.71 | 0.54 | 0.055 |
| **Median calendar year of BC diagnosis** | | | | | | | |
| >2008 | 5 | 3611 | 16546 | 1.03 | 0.79, 1.35 | 0.00 | 0.650 |
| ≤2008 | 8 | 2541 | 8185 | 1.22 | 0.88, 1.68 | 0.61 | 0.012 |
| **Sample size** | | | | | | | |
| >1000 | 3 | 5173 | 23094 | 1.04 | 0.93, 1.16 | 0.18 | 0.297 |
| ≤1000 | 10 | 979 | 1637 | 1.17 | 0.84, 1.63 | 0.53 | 0.025 |
| **Proportion of young participants** | | | | | | | |
| >25 | 4 | 424 | 387 | 1.34 | 0.94, 1.91 | 0.00 | 0.742 |
| ≤25 | 6 | 5441 | 23924 | 1.09 | 0.75, 1.60 | 0.70 | 0.005 |
| unknown | 3 | 287 | 420 | 1.03 | 0.59, 1.78 | 0.00 | 0.382 |
| **BMI** | | | | | | | |
| adjusted | 2 | 234 | 187 | 1.64 | 0.90, 2.97 | 0.00 | 0.753 |
| not adjusted | 11 | 5918 | 24544 | 1.09 | 0.85, 1.40 | 0.50 | 0.028 |
| **SES** | | | | | | | |
| adjusted | 3 | 4731 | 21470 | 0.97 | 0.69, 1.37 | 0.00 | 0.512 |
| not adjusted | 10 | 1421 | 3261 | 1.21 | 0.92, 1.58 | 0.48 | 0.043 |
| **Comorbidity score** | | | | | | | |
| adjusted | 2 | 4711 | 21450 | 1.00 | 0.92, 1.08 | 0.00 | 0.639 |
| not adjusted | 11 | 1441 | 3281 | 1.17 | 0.88, 1.57 | 0.47 | 0.040 |
| **DM determined by metformin** | | | | | | | |
| no | 11 | 5969 | 24071 | 1.20 | 0.96, 1.51 | 0.36 | 0.108 |
| yes | 2 | 183 | 660 | 0.74 | 0.36, 1.55 | 0.57 | 0.125 |
| **Time window ≥1 year** | | | | | | | |
| no | 11 | 5806 | 24020 | 1.17 | 0.94, 1.45 | 0.27 | 0.186 |
| yes | 2 | 346 | 711 | 0.95 | 0.32, 2.78 | 0.86 | 0.007 |
| **Overall quality** | | | | | | | |
| high | 2 | 1695 | 5532 | 1.20 | 0.77, 1.88 | 0.49 | 0.160 |
| low/median | 11 | 4457 | 19199 | 1.11 | 0.85, 1.45 | 0.51 | 0.026 |
| **ROB of study participation** | | | | | | | |
| low | 2 | 1695 | 5532 | 1.20 | 0.77, 1.88 | 0.49 | 0.160 |
| median/high | 11 | 4457 | 19199 | 1.11 | 0.85, 1.45 | 0.51 | 0.026 |

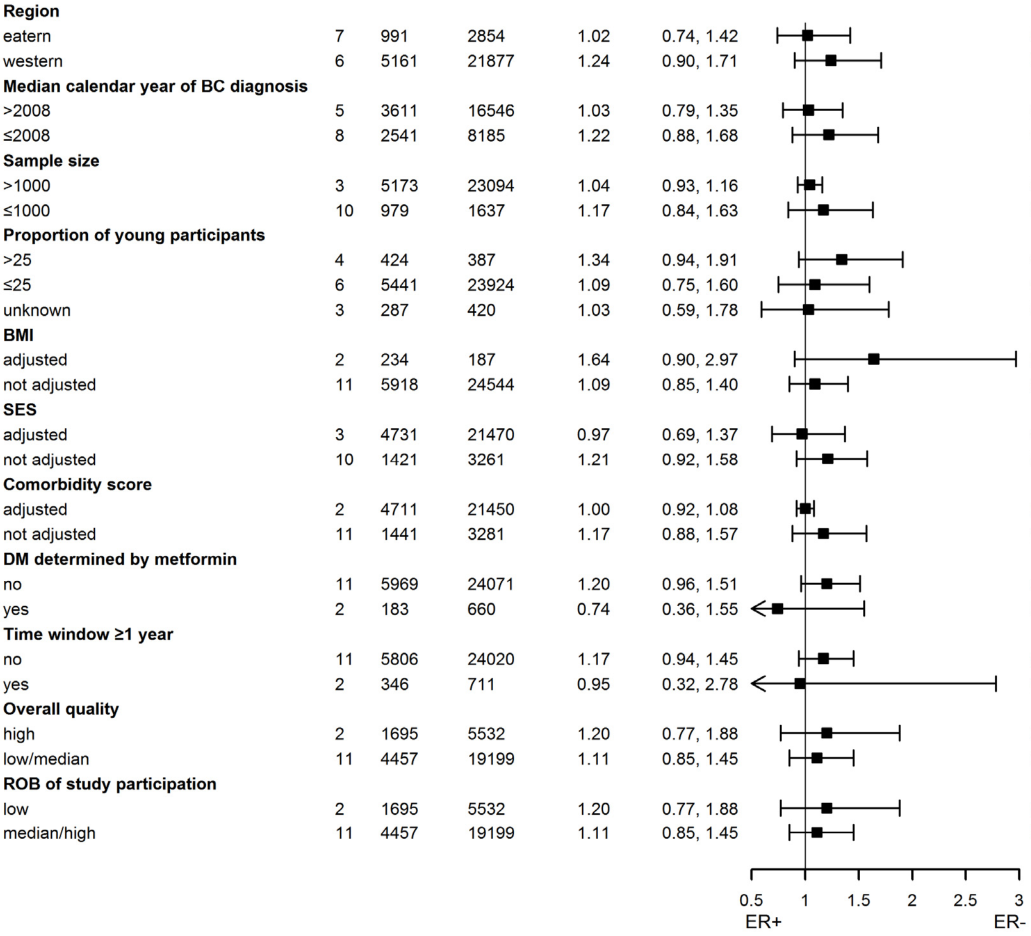


**Figure S3.** Stratified analyses to find possible interpretation for heterogeneity in the association between T2DM and ER status. N: the number of studies; T2DM: type 2 diabetes mellitus; BC: breast cancer; DM, diabetes mellitus; ER: estrogen receptor; BMI: body Mass index; SES: socioeconomic status; pORs: pooled odds ratios; CI, confidence interval; ROB: risk of bias; P_Qtest: *P*-value from Q-test.

**PR status**

| Study characteristics | N | Sample sizes T2DM | Sample sizes non-T2DM | pORs | 95% CI | I-squared | P_Qtest |
|---|---|---|---|---|---|---|---|
| **Region** | | | | | | | |
| eatern | 7 | 1035 | 2852 | 1.02 | 0.72, 1.46 | 0.59 | 0.024 |
| western | 5 | 1913 | 5650 | 1.14 | 0.77, 1.70 | 0.78 | 0.001 |
| **Median calendar year of BC diagnosis** | | | | | | | |
| >2008 | 4 | 384 | 529 | 0.92 | 0.66, 1.28 | 0.00 | 0.505 |
| ≤2008 | 8 | 2564 | 7973 | 1.16 | 0.83, 1.63 | 0.78 | <0.001 |
| **Sample size** | | | | | | | |
| >1000 | 2 | 1925 | 6867 | 0.89 | 0.66, 1.21 | 0.84 | 0.014 |
| ≤1000 | 10 | 1023 | 1635 | 1.14 | 0.84, 1.56 | 0.62 | 0.004 |
| **Proportion of young participants** | | | | | | | |
| >25 | 4 | 470 | 389 | 1.55 | 1.10, 2.17 | 0.00 | 0.716 |
| ≤25 | 5 | 2192 | 7691 | 1.00 | 0.65, 1.54 | 0.81 | <0.001 |
| unknown | 3 | 286 | 422 | 0.81 | 0.59, 1.11 | 0.00 | 0.932 |
| **BMI** | | | | | | | |
| adjusted | 2 | 235 | 187 | 1.45 | 0.87, 2.40 | 0.00 | 0.928 |
| not adjusted | 10 | 2713 | 8315 | 1.03 | 0.77, 1.38 | 0.71 | <0.001 |
| **SES** | | | | | | | |
| adjusted | 2 | 1483 | 5243 | 0.77 | 0.66, 0.89 | 0.00 | 0.815 |
| not adjusted | 10 | 1465 | 3259 | 1.15 | 0.87, 1.52 | 0.62 | 0.005 |
| **DM determined by metformin** | | | | | | | |
| no | 10 | 2766 | 7848 | 1.15 | 0.88, 1.50 | 0.70 | <0.001 |
| yes | 2 | 182 | 654 | 0.72 | 0.39, 1.33 | 0.41 | 0.191 |
| **Time window ≥1 year** | | | | | | | |
| no | 10 | 2602 | 7797 | 1.12 | 0.85, 1.46 | 0.68 | 0.001 |
| yes | 2 | 346 | 705 | 0.90 | 0.38, 2.09 | 0.84 | 0.014 |
| **Overall quality** | | | | | | | |
| high | 2 | 1674 | 5324 | 0.98 | 0.56, 1.73 | 0.78 | 0.035 |
| low/median | 10 | 1274 | 3178 | 1.10 | 0.82, 1.49 | 0.61 | 0.006 |
| **ROB of study participation** | | | | | | | |
| low | 2 | 1674 | 5324 | 0.98 | 0.56, 1.73 | 0.78 | 0.035 |
| median/high | 10 | 1274 | 3178 | 1.10 | 0.82, 1.49 | 0.61 | 0.006 |

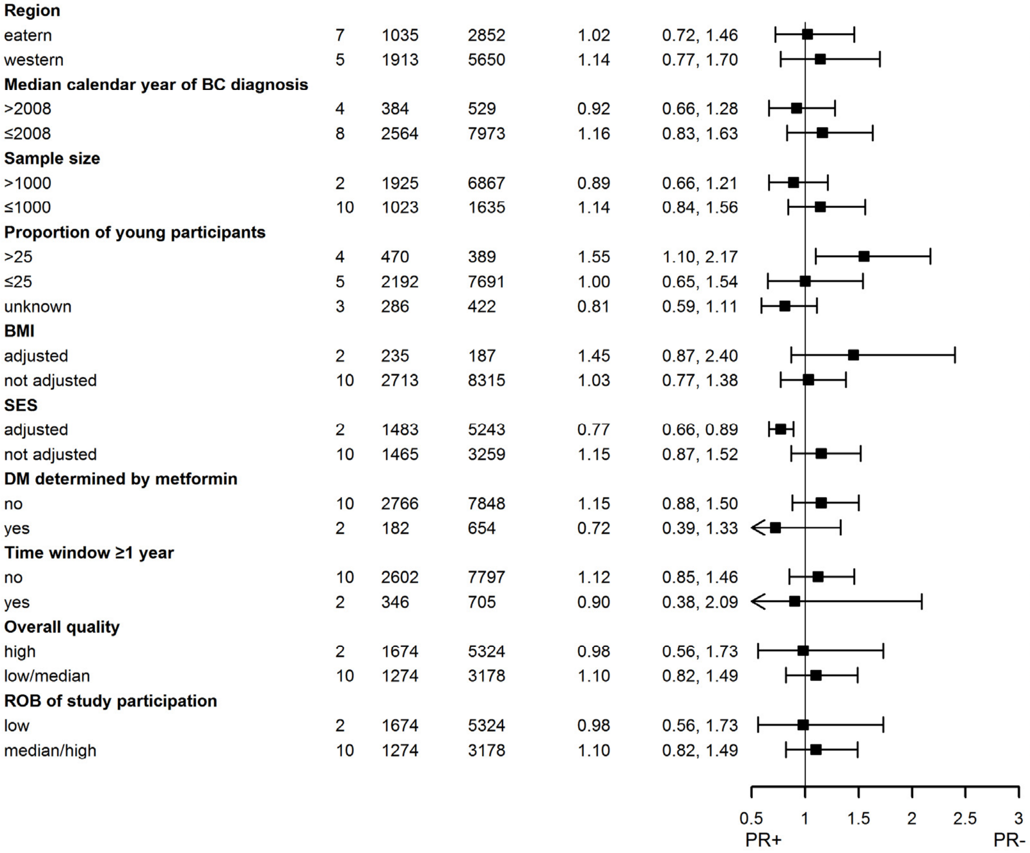


**Figure S4.** Stratified analyses to find possible interpretation for heterogeneity in the association between T2DM and PR status. N: the number of studies; T2DM: type 2 diabetes mellitus; BC: breast cancer; DM, diabetes mellitus; PR: progesterone receptor; BMI: body Mass index; SES: socioeconomic status; pORs: pooled odds ratios; CI, confidence interval; ROB: risk of bias; P_Qtest: $P$-value from Q-test.

**Her2 status**

| Study characteristics | N | Sample T2DM | sizes non-T2DM | pORs | 95% CI | I-squared | P_Qtest |
|---|---|---|---|---|---|---|---|
| **Region** | | | | | | | |
| eatern | 6 | 960 | 2773 | 0.90 | 0.63, 1.28 | 0.48 | 0.088 |
| western | 5 | 1716 | 4498 | 0.75 | 0.44, 1.27 | 0.54 | 0.069 |
| **Median calendar year of BC diagnosis** | | | | | | | |
| >2008 | 3 | 360 | 489 | 1.25 | 0.89, 1.77 | 0.00 | 0.592 |
| ≤2008 | 8 | 2316 | 6782 | 0.73 | 0.54, 0.98 | 0.54 | 0.034 |
| **Sample size** | | | | | | | |
| >1000 | 2 | 1730 | 5717 | 1.02 | 0.84, 1.25 | 0.32 | 0.226 |
| ≤1000 | 9 | 946 | 1554 | 0.77 | 0.53, 1.11 | 0.49 | 0.048 |
| **Proportion of young participants** | | | | | | | |
| >25 | 4 | 420 | 391 | 0.67 | 0.39, 1.18 | 0.53 | 0.093 |
| ≤25 | 5 | 1994 | 6498 | 0.82 | 0.56, 1.20 | 0.49 | 0.098 |
| unknown | 2 | 262 | 382 | 1.23 | 0.78, 1.92 | 0.00 | 0.351 |
| **BMI** | | | | | | | |
| adjusted | 2 | 233 | 187 | 0.47 | 0.25, 0.88 | 0.00 | 0.724 |
| not adjusted | 9 | 2443 | 7084 | 0.91 | 0.67, 1.23 | 0.45 | 0.071 |
| **DM determined by metformin** | | | | | | | |
| no | 9 | 2497 | 6660 | 0.90 | 0.66, 1.22 | 0.45 | 0.067 |
| yes | 2 | 179 | 611 | 0.57 | 0.25, 1.28 | 0.54 | 0.140 |
| **Time window ≥1 year** | | | | | | | |
| no | 9 | 2339 | 6610 | 0.87 | 0.61, 1.24 | 0.50 | 0.043 |
| yes | 2 | 337 | 661 | 0.67 | 0.42, 1.07 | 0.00 | 0.367 |
| **Overall quality** | | | | | | | |
| high | 2 | 1477 | 4174 | 0.77 | 0.45, 1.32 | 0.56 | 0.130 |
| low/median | 9 | 1199 | 3097 | 0.85 | 0.60, 1.22 | 0.52 | 0.034 |
| **ROB of study participation** | | | | | | | |
| low | 2 | 1477 | 4174 | 0.77 | 0.45, 1.32 | 0.56 | 0.130 |
| median/high | 9 | 1199 | 3097 | 0.85 | 0.60, 1.22 | 0.52 | 0.034 |

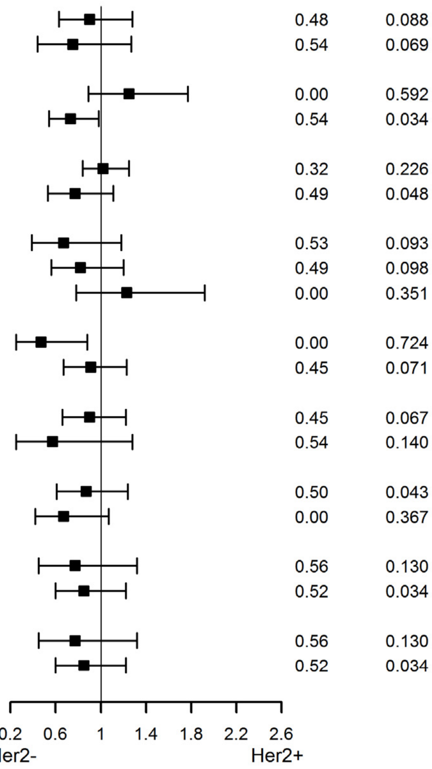


**Figure S5.** Stratified analyses to find possible interpretation for heterogeneity in the association between T2DM and Her2 expression. N: the number of studies; T2DM: type 2 diabetes mellitus; BC: breast cancer; DM, diabetes mellitus; Her2: human epidermal growth factor receptor-2; BMI: body Mass index; pORs: pooled odds ratios; CI, confidence interval; ROB: risk of bias; P_Qtest: *P*-value from Q-test.

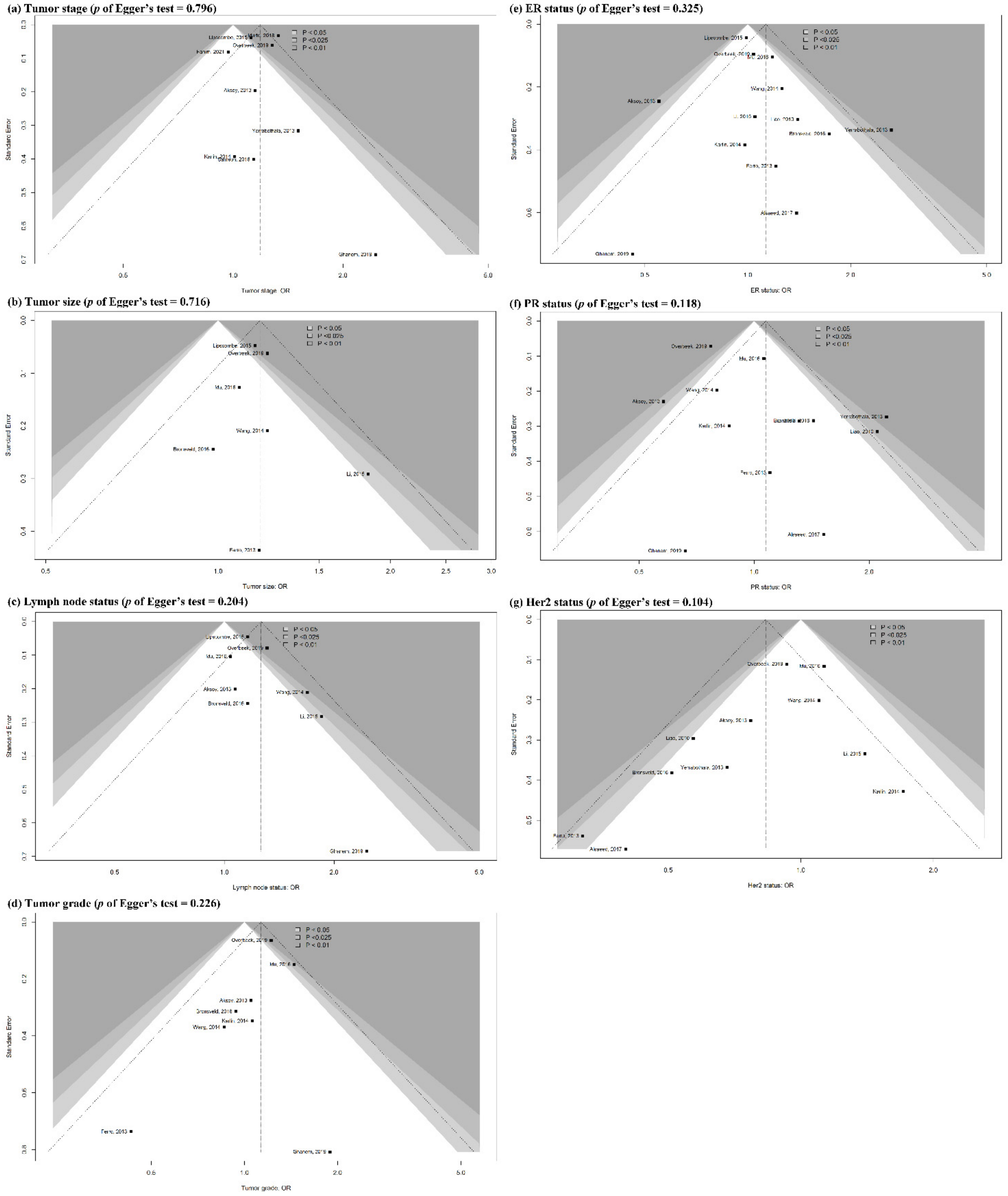


**Figure S6.** Funnel plots for T2DM and breast tumor characteristics. Funnel plots were created for (a) tumor stage; (b) tumor size; (c) lymph node status; (d) tumor grade; (e) ER status; (f) PR status; (g) Her2 status. ER: estrogen receptor; PR: progesterone receptor; Her2: human epidermal growth factor receptor-2; OR: odds ratio.

**Table S1.** Additional characteristics of studies included in the systematic review and meta-analysis.

| First author (year) | Menopausal status (number) | | Inclusion and Exclusion criteria | Ascertainment of DM | |
|---|---|---|---|---|---|
| | breast cancer with DM | breast cancer without DM | | Identification of DM patients | T1DM |
| Fahim (2021) | no information | | **Inclusion:**<br>- women with newly diagnosed BC between 2008 and 2013;<br>- continuously enrolled in Medicare Parts A, B, and D for 12 months before their cancer diagnosis;<br>**Exclusion:**<br>- enrolled in managed care;<br>- unknown diagnosis stages or without using any medications within 1 year before cancer diagnosis. | KPWA automated electronic health records and claims data: having 1+ inpatient or 2+ outpatient ICD-9 diagnosis codes for diabetes; any diabetes medication use (insulin and oral medications); HbA1c ≥7%, 1 FBG ≥200mg/mL, or 2 FBG ≥126 mg/mL in the 2 years prior to BC diagnosis. | not mentioned. |
| Overbeek (2019) | no information | | **Inclusion:**<br>- women diagnosed with invasive BC (stages I–IV);<br>- at least 4 years of continuous enrollment in the PHARMO Database Network prior to the diagnosis of BC;<br>**Exclusion:**<br>- women with a history of oophorectomy prior to the diagnosis of BC. | the PHARMO Database Network: receiving two or more dispensings of noninsulin blood glucose–lowering drugs (NIBGLDs) (ATC code A10B) within 6 months in the 4 years prior to their diagnosis of BC. | excluded. |
| Chen (2019) | pre-, post-menopause:<br>ER+/HER2-: 962, 999;<br>ER+/HER2+: 200, 113;<br>TNBC: 124; 647, 769;<br>H2E: 248, 320. | | **Inclusion:**<br>- newly diagnosed invasive BC;<br>- aged 20 to 69 at the time of BC diagnosis;<br>- with complete tumor marker (ER/PR/Her2) information;<br>**Exclusion:**<br>- with missing diabetes history data. | medical records (diabetes history and use of common diabetes medications) and patient self-reports. | excluded [1]. |
| Ghanem (2019) | no information. | | **Inclusion:**<br>- having primary invasive ductal BC;<br>- having received mammary gland surgery.<br>**Exclusion:**<br>- histologically ductal carcinoma in situ, lobular carcinoma in situ;<br>- patients with clinical manifestations of infections. | medical examination: the American Diabetes Association (2013). | excluded. |
| Murto (2018) | no information. | | **Inclusion:**<br>- newly diagnosed BC women in Finland;<br>**Exclusion:**<br>- carcinoma in situ only;<br>- missing date of later diagnosis of invasive cancer. | the Finnish Care Register for Health Care (HILMO): DM diagnosis, and the Finnish Social Insurance Institution (SII): yearly drug purchases. | not mentioned. |
| Alsaeed (2017) | pre-, post-menopause: 91; 19. | | **Inclusion:**<br>- women with histopathologically confirmed BC;<br>- having either breast conserving surgery with axillary lymph node dissection or modified radical mastectomy after chemotherapy and radiotherapy | FBG >126mg/dL. | not mentioned. |

| | | | | | |
|---|---|---|---|---|---|
| | | | **Exclusion:**<br>- incompletion of the above listed treatment regimen. | | |
| Mu (2017) | no information. | | **Inclusion:**<br>- BC patients;<br>**Exclusion:**<br>- with T1DM;<br>- diagnosed as diabetes when BC diagnosis;<br>- without complete medical records;<br>- with a history of sever hypoglycemia;<br>- treated with both insulin and other anti-diabetic agents after BC diagnosis within 5 years;<br>- dying from other causes within 5 years of BC diagnosis and lost follow up. | medical and medication history and the blood glucose monitoring status from by physical examinations. | excluded. |
| Bronsveld (2016) | pre-, post-menopause: 110, 101. | pre-, post-menopause: 49, 52. | **Inclusion:**<br>- women with diabetes in strata of age ≤50 and >50 years (1:1) at BC diagnosis;<br>- matched women without diabetes at BC diagnosis;<br>**Exclusion:**<br>- with a history of other cancers, non-invasive or metastasized BC;<br>- treated with neoadjuvant therapy;<br>- patients with diabetes diagnosed ≤1 year prior to their BC diagnosis;<br>- patients with no or insufficient tumor tissue. | the National Patient Register: DM diagnosis; and the Danish Register of Medicinal Products Statistics diagnosis: medication use, and electronic patient files: HbA1C levels prior to BC diagnosis. | not excluded [2]. |
| Samson (2016) | pre-, post-menopause:<br>African American: 310, 369;<br>European American: 172, 520. | | **Inclusion:**<br>- African American and European American women;<br>- a BC diagnosis in both the SCCCR ICD-O and Medicaid ICD-9 designations.<br>**Exclusion:**<br>- previous BC diagnosis. | Medicaid records: a diagnosis of T2DM or if a woman filled a prescription for a drug used for treating diabetes. | excluded. |
| Lipscombe (2015) | no information. | | **Inclusion:**<br>- Ontario women aged 20–105 years;<br>- newly diagnosed with invasive BC;<br>- valid cancer stage information;<br>- at least 1 year of health services data prior to diagnosis.<br>**Exclusion:**<br>- any other previous cancer (except for non-melanoma skin cancer) or carcinoma in situ tumors. | the validated Ontario Diabetes Database: DM diagnosis prior to BC diagnosis. | not mentioned. |
| Li (2015) | pre-, post-menopause: 25, 73. | pre-, post-menopause: 30, 77. | **Inclusion:**<br>- histologically confirmed female invasive ductal BC;<br>- without any chemo- or radio-therapy before surgery. | WHO 1999. | excluded. |
| Karlin (2014) | no information. | | **Inclusion:**<br>- newly diagnosed BC. | electronic medical records; glucose and HbA1c data for the study period from the laboratory information system. | not excluded [3]. |
| Wang (2014) | no information. | | **Inclusion:**<br>- diagnosed with primary BC;<br>**Exclusion:**<br>- T1DM; | the medical and medication history, and the blood glucose monitoring status from physical examinations (criteria for diabetes diagnosis: WHO 1999). | excluded. |

| | | | | | |
|---|---|---|---|---|---|
| | | | - with history of cancer;<br>- with bilateral or metastatic disease at the time of diagnosis;<br>- male patient. | | |
| Aksoy (2013) | pre-, peri-, post-meno-pause: 17, 12, 119. | pre-, peri-, post-meno-pause: 167, 250, 219. | **Inclusion:**<br>- women with BC;<br>**Exclusion:**<br>- receiving insulin treatment;<br>- diagnosed with diabetes mellitus within 12 months before BC diagnosis. | taking metformin for diabetes mellitus. | excluded. |
| Ferro (2013) | no information. | | **Inclusion:**<br>- female BC patients treated with concurrent metformin and radiotherapy;<br>- matched nondiabetic BC patients. | taking metformin for diabetes mellitus. | excluded. |
| Yerrabothala (2013) | no information. | | **Inclusion:**<br>- diagnosed with BC;<br>**Exclusion:**<br>- BC patients with a decreased survival secondary to non-BC-related malignancy, familial BC;<br>- patients diagnosed with DM after diagnosis of BC. | medical records: anti-diabetic treatment and HbA1c. | excluded. |
| Liao (2010) | pre-, post-menopause: 69, 74. | pre-, post-menopause: 115, 28. | **Inclusion:**<br>- female patients aged 29 to 77;<br>- BC diagnosed by pathological examination;<br>- having complete data and systemic treatment.<br>**Exclusion:**<br>- with other malignant tumor. | routine laboratory evaluation: FBG >7.1 mmol/L. | excluded. |

[1] patients with T1DM or gestational diabetes, but without a history of T2DM were categorized as unexposed; [2] T1DM patients ($n$ =25); [3] number of T2DM/T1DM: 96/13; DM: diabetes mellitus; BC: breast cancer; T1DM: Type 1 diabetes mellitus; T2DM: Type 2 diabetes mellitus; FBG: fasting blood glucose; ER: estrogen receptor; PR: progesterone receptor; Her2: human epidermal growth factor receptor-2; H2E: ER-/PR-/Her2+; TNBC: triple-negative breast cancer.

**Table S2.** Methodological quality of the included studies based on the QUIPS tool.

| Study Characteristics | | Risk of bias based on QUIPS | | | | | | |
|---|---|---|---|---|---|---|---|---|
| **First author (year)** | **Study design** | **1. study participation** | **2. Study Attrition** | **3. Diabetes Measurement** | **4. Outcome Measurement** | **5. Study Confounding** | **6. Statistical Analysis and Reporting** | **Overall quality** |
| Fahim (2021) | CS | ** | n/a | ** | * | ** | * | ** |
| Overbeek (2019) | nested CC | * | * | ** | * | ** | * | * |
| Chen (2019) | CC | ** | n/a | * | * | * | * | ** |
| Ghanem (2019) | CS | *** | n/a | ** | * | ** | ** | *** |
| Murto (2018) | CS | * | n/a | ** | * | * | * | * |
| Alsaeed (2017) | CS | ** | n/a | *** | * | ** | *** | *** |
| Mu (2017) | CS | *** | n/a | * | * | ** | * | *** |
| Bronsveld (2016) | CS | * | n/a | * | * | * | * | * |
| Samson (2016) | CS | *** | n/a | ** | * | ** | * | *** |
| Lipscombe (2015) | CS | ** | n/a | * | * | ** | * | ** |
| Li (2015) | CS | *** | n/a | ** | ** | ** | * | *** |
| Karlin (2014) | CS | *** | n/a | * | * | ** | * | *** |
| Wang (2014) | CS | ** | n/a | * | * | ** | * | ** |
| Aksoy (2013) | CS | ** | n/a | *** | * | ** | * | *** |
| Ferro (2013) | CS | ** | n/a | *** | ** | ** | ** | *** |
| Yerrabothala (2013) | CS | ** | n/a | ** | * | ** | * | ** |
| Liao (2010) | CS | *** | n/a | ** | ** | ** | *** | *** |

*: low risk of bias or high quality; **: moderate risk of bias or moderate quality; ***: high risk of bias or low quality; n/a: not applicable; CC: case-control; CS: cross-sectional.

**Table S3.** Sensitivity analyses.

| **Tumor characteristics** | **Comparisons** | **Sample sizes** | | **# of studies** | **pooled OR** | **95% CI** | ***P* values** |
|---|---|---|---|---|---|---|---|
| | | Non-T2DM | T2DM | | | | |
| tumor stage | stage IV versus cancers in situ & stage I–III | 95922 | 12946 | 6 | 1.24 | 1.05, 1.47 | 0.011 |
| | stage III–IV versus stage I–II (excluding papers with in situ tumors) | 95366 | 12813 | 6 | 1.23 | 1.05, 1.44 | 0.010 |
| | stage III–IV versus cancers in situ & stage I–II (only including papers with tumors at stage IV) | 102109 | 14767 | 8 | 1.18 | 1.07, 1.31 | 0.001 |
| tumor size | ≥50 mm versus <50 mm | 8576 | 2585 | 8 | 1.30 | 0.96, 1.76 | 0.095 |
| lymph node status | N2–3 versus N0–1 | 6933 | 2077 | 5 | 1.98 | 0.75, 5.23 | 0.165 |
| tumor grade | grade 3 versus grade 1–2 | 6216 | 1952 | 8 | 1.25 | 0.91, 1.70 | 0.165 |

T2DM: Type 2 diabetes mellitus; OR, odds ratio; CI, confidence interval; *P* values: from *Z* test to test the pooled OR.

## References


1. Hamling, J.; Lee, P.; Weitkunat, R.; Ambuhl, M. Facilitating meta-analyses by deriving relative effect and precision estimates for alternative comparisons from a set of estimates presented by exposure level or disease category. *Stat. Med.* **2008**, *27*, 954–970, doi:10.1002/sim.3013.
2. Bronsveld, H.K.; Jensen, V.; Vahl, P.; De Bruin, M.L.; Cornelissen, S.; Sanders, J.; Auvinen, A.; Haukka, J.; Andersen, M.; Vestergaard, P.; et al. Diabetes and Breast Cancer Subtypes. *PLoS One* **2017**, *12*, e0170084, doi:10.1371/journal.pone.0170084.
